# Chemical Variability and Biological Activities of *Eucalyptus* spp. Essential Oils

**DOI:** 10.3390/molecules21121671

**Published:** 2016-12-07

**Authors:** Luiz Claudio Almeida Barbosa, Claudinei Andrade Filomeno, Robson Ricardo Teixeira

**Affiliations:** 1 Departament of Chemistry, ICEx, Universidade Federal de Minas Gerais, Av. Presidente Antônio Carlos, 6627, Campus Pampulha, Belo Horizonte, MG 31270-901, Brazil; lcab@ufmg.br; 2 Departament of Chemistry, Universidade Federal de Viçosa, Av. P.H. Rolfs, S/N, Viçosa, MG 36570-900, Brazil; robsonr.teixeira@ufv.br; 3 Instituto Federal do Espírito Santo, Av. Ministro Salgado Filho, 1000, Campus Vila Velha, Vila Velha, ES 29106-010, Brazil

**Keywords:** essential oils, monoterpenes, insecticidal activity, antimicrobial activity, acaricidal activity, herbicidal activity, *Eucalyptus*, 1,8-cineole

## Abstract

Many plant species produce mixtures of odorous and volatile compounds known as essential oils (EOs). These mixtures play important roles in Nature and have been utilized by mankind for different purposes, such as pharmaceuticals, agrochemicals, aromatherapy, and food flavorants. There are more than 3000 EOs reported in the literature, with approximately 300 in commercial use, including the EOs from *Eucalyptus* species. Most EOs from *Eucalyptus* species are rich in monoterpenes and many have found applications in pharmaceuticals, agrochemicals, food flavorants, and perfumes. Such applications are related to their diverse biological and organoleptic properties. In this study, we review the latest information concerning the chemical composition and biological activities of EOs from different species of *Eucalyptus*. Among the 900 species and subspecies of the *Eucalyptus* genus, we examined 68 species. The studies associated with these species were conducted in 27 countries. We have focused on the antimicrobial, acaricidal, insecticidal and herbicidal activities, hoping that such information will contribute to the development of research in this field. It is also intended that the information described in this study can be useful in the rationalization of the use of *Eucalyptus* EOs as components for pharmaceutical and agrochemical applications as well as food preservatives and flavorants.

## 1. Introduction

Nature is a precious reservoir of substances that can be explored for developing new pharmaceuticals. Several drugs for treating a variety of diseases have been discovered via screening of natural compounds obtained from animals, microorganisms, marine organisms, and plants. These drugs can be natural products *per se* or semi-synthetic analogs derived from an active natural product. Furthermore, they can be entirely synthetic compounds designed using natural products as models [1,2,3,4,5,6].

Natural products have also been directly utilized as pest control agents. Moreover, they have served as models for the development of new pesticides with potential commercial applications [7,8,9,10,11,12,13].

Although there are a large number of plant species, only approximately 10% produce mixtures of odorous and volatile compounds, collectively called essential or volatile oils [14]. Such essential oils (EOs) can be produced from all parts of plants (buds, gums, blossoms, flowers, leaves, stems, twigs, seeds, fruits, roots, wood or bark), depending upon the producing species. EOs are stored in several secretory structures such as epidermic cells, secretory hairs, secretory ducts, secretory cavities, glandular trichomes, or resin adducts [15,16,17,18,19]. EOs are generally hydrophobic liquids and soluble in alcohol, nonpolar or weakly polar solvents, waxes, and oils. They are slightly soluble in water and are usually colorless or pale yellow [15,17,20]. From a chemical standpoint, they are typically composed of hydrocarbons and oxygenated monoterpenes, sesquiterpenes and diterpenes, aromatic compounds (C6-C3 and C6-C1 compounds), and low molecular weight aliphatic compounds.

Some EOs play an important role in protecting plants against insect attack, fungi, bacteria and viruses and can also be important as a deterrent to herbivorous feeding [15,21,22,23,24]. EOs are also known to be involved in allelopathic interactions inhibiting seed germination and plant growth [25,26,27,28]. These properties have been investigated for the development of herbicides [29,30,31]. Within this context and considering the favorable biodegradability of essential oil components, they can be considered attractive alternative tools for controlling the growth of weeds [32]. EOs from a variety of plants are also endowed with antibacterial activities [33,34,35,36] as well as anti-inflammatory and antioxidant properties [37].

There are more than 3000 EOs described in the existing literature, with approximately 300 in commercial use, including those from various *Eucalyptus* species [15,17,38,39]. The *Eucalyptus* genus is represented by 900 species and subspecies. Based on morphological and molecular characteristics, *Eucalyptus* was reclassified in 1995 by Hill and Johnson [40]. According these authors, the *Corymbia*, previously classified as a subgenus of *Eucalyptus*, has been elevated to the rank of a separate genus with 113 known *Corymbia* species. Among then, *Corymbia citriodora*, *C. maculata*, *C. ficifolia*, *C. ptychocarpa* and *C. torelliana* are the best-known. Despite this reclassification, the names originally found in the references were used for the preparation of this review to facilitate the discussion.

The *Eucalyptus* corresponds to one of the principal genera of the Myrtaceae family, native to Australia and cultivated worldwide [17,41,42,43,44]. *Eucalyptus* trees have perennial leaves that are odorous because of the presence of EOs that are produced and stored in secretory cells. These EOs are aromatic, spicy, and colorless or pale yellow, although there are studies that have reported the color as being brownish or greenish [44].

EOs obtained from *Eucalyptus* are usually rich in monoterpenes and in some cases sesquiterpenes. Many such EOs are used for pharmaceutical purposes and in perfumery [45]. The eucalyptus EOs utilized as pharmaceuticals are rich in 1,8-cineole, whereas those used in perfumery are rich in citronellal, citral and geranyl acetate [46].

Considering the versatility of *Eucalyptus* EOs in terms of bioactivities as well as their industrial importance, the purpose of this study is to provide the readers with the latest information concerning the chemical composition and biological activities of EOs from different species of *Eucalyptus*. Two reviews about *Eucalyptus* EOs and biological activities have been recently published. One of them by Vuong et al. [47] that focused on anticancer properties of *Eucalyptus* EOs; the other by Zhang et al. [48] that described advances up to 2010 in terms of several biological activities. In this paper, from the 900 species and subspecies of the *Eucalyptus* genus, we have examined 68 species (three of them are hybrids). The studies associated with these species were conducted in 27 countries and the literature survey covers recent developments in the field. The review focused on the antimicrobial, acaricidal, insecticidal and herbicidal properties of *Eucalyptus* species. The information described can be useful in the rationalization of the use of *Eucalyptus* EOs as source of constituents for pharmaceutical and agrochemical applications as well as food preservatives.

## 2. Chemical Variability of *Eucalyptus* EOs

**Figure 1:**
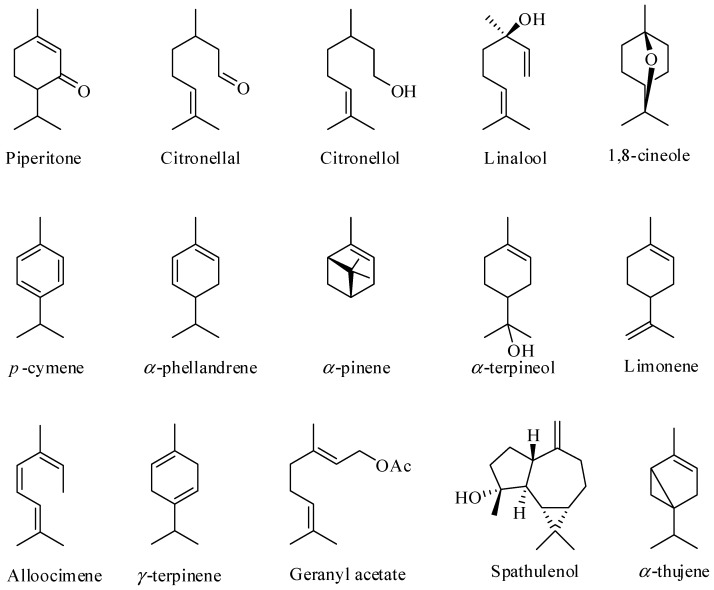
Some of the major constituents of the essential oils of *Eucalyptus* leaves.

Although the EOs are found in the leaves of more than 300 species of *Eucalyptus*, fewer than 20 species have been commercially explored for EO production [46,49]. In terms of the chemical composition of these EOs, they are complex mixtures of substances, generally containing 20 to 80 compounds, differing in their concentrations. Terpenes and terpenoids are the major components found in EOs obtained from the leaves of *Eucalyptus* [38,50,51,52,53,54,55] as illustrated in Figure 1.

The International Standard Organization (ISO) defines EOs as products obtained from parts of plants through hydrodistillation, steam distillation or dry distillation, as well as products obtained by a suitable mechanical process (for *Citrus* fruits). The definition of an essential oil excludes other aromatic/volatile products obtained by different extractive techniques such as extraction with solvents (concretes, absolutes), supercritical fluid extraction, and microwave-assisted extraction.

The composition of the EOs can vary according to the method and drying conditions applied to the vegetal material prior to extraction, and also according to the storage conditions [56,57,58,59]. The method of choice for a particular application depends on the material from which the EOs are to be extracted and also the type of application itself.

Concerning the extraction of EOs from *Eucalyptus*, hydrodistillation is typically the method of choice. The extraction yields range from 0.06% to 7% [60], and the chemical composition of the resulting EOs depends on the plant species and varieties. Within the same variety, the essential oil composition can vary according to geographical region, as reported in several studies [15,17,39,61,62] (Table 1).

From Table 1, it can be noticed that the species *E. camaldulensis*, *E. cinerea*, *E. citriodora*, *E. globulus*, *E. grandis*, *E. saligna* and *E. tereticornis* are the ones which have received more attention in terms of their essential oil composition. A more detailed discussion regarding chemical aspects of EOs of these species is described below.

### 2.1. Eucalyptus camaldulensis Dehnh

The reported yields of EOs for *E. camaldulensis* range from 0.26% to 3.48% being the highest value found for plants cultivated in Taiwan [77]. In most *E. camaldulensis* EOs, 1,8-cineole is the major constituent, usually found in quantities above 50% in EOs extracted from plants cultivated in Egypt [69], the Democratic Republic of the Congo [53], Nigeria [73], Brazil [66,67] and Iran [71]. Different chemotypes of *E. camaldulensis* were identified for plants cultivated in Spain and Taiwan. Plants from Spain showed spathulenol and *p*-cymene as the major components [76], while for the species from Taiwan the principal constituents were α-pinene, *p*-cymene and α-phellandrene [78]. Plants cultivated in different countries produces EOs with variable composition as can be seen from Table 1.

### 2.2. Eucalyptus cinerea F. Muell. ex Benth

The leaves of *E. cinerea* are aromatic, with great potential for EO production, and are used for ornamental purposes. There are few reports of its use in folk medicine [135]. Among all herein described *Eucalyptus* species, *E. cinerea* is the one that produces the highest amount of EOs, as illustrated by plants cultivated in Argentina that afford 2.48% [63,64] and those from Paraná state in Brazil with 6.07% [85]. As observed from Table 1, the EOs produced by *E. cinerea* usually contain more than 80% of 1,8-cineole [81,84] and such oils may serve as a source of this important compound for industrial applications.

### 2.3. Eucalyptus citriodora Hook

The EOs extracted from *E. citriodora* is the most important in terms of worldwide trading volume [99,136]. This species constitutes the richest and most economical known source of citronellal, a substance widely used in the manufacture of cosmetics and aromatization of cleaning products such as soaps and detergents. This compound also has antiseptic properties, which justifies its use as a cleaning agent and disinfectant of floors and toilets [137]. In terms of chemical composition, the EOs produced by *E. citriodora* are the most widely investigated among all eucalyptus species. In general, this species affords high yields of EOs, as observed in the studies from some plants cultivated in India [54] and in Benin [89] (4.8% and 4.6% yields, respectively). Lower EOs yielding species were found, however, among plants cultivated in India (0.6%) [26], in São Paulo state, Brazil (0.66%) [66,67] and Colombia (0.70%) [103].

As observed from the data presented in Table 1, plants cultivated in several states in Brazil usually produce EOs with high (>70%) citronellal content [66,67,91,92,93,94,95,96]. Other examples of plants that produce EOs with citronellal content above 70% are those from the Democratic Republic of the Congo [53]; South Korea [107] and Argentina [82]. Analysis of the data presented in Table 1 reveals that yields of EOs produced by these species and also their citronellal contents are influenced by the plant cultivation location.

As reported to date, only plants cultivated in Tunisia [49,108] and Kenya [72] do not present citronellal as the major component in their EOs. Therefore, these *E. citriodora* species represent different chemotypes producing EOs rich in 1,8-cineole and α-pinene (the Tunisian species) and 1,8-cineole for species cultivated in Kenya.

### 2.4. Eucalyptus globulus Labill

The EOs produced by *E. globulus*, cultivated in several places, are the major commercial source of 1,8-cineole. The highest content of 1,8-cineole (>80%) in EOs of *E. globulus* was reported in studies carried out in Brazil in São Paulo state [114,115], in Minas Gerais state [91], and in Ceará state [95]. High 1,8-cineole content was also found in EOs from Australia (81.1%–90.0%) [112,113]; Indonesia (86.5%) [106]; Montenegro (85.8%) [126]; Italy (84.9%) [125]; India (81.9%) [119]; Iran [123].

A severe limitation on several studies with *E. globulus* EOs is the lack of information on the extraction yields. This fact precludes us from evaluating the potential commercial application of such plants as a source of 1,8-cineole. Therefore, the plants that produce EOs with high 1,8-cineole content should be further investigated in more details in case of a commercial interest.

### 2.5. Eucalyptus grandis W. Hill ex Maiden

As described for other eucalyptus species, different chemotypes were also reported for *E. grandis*. Thus, plants cultivated in Goiás state (Brazil) are representative of chemotypes with γ-terpinene, *o*-cymene and β-pinene as the major components of their EOs [93]. In another study conducted in Botucatu (São Paulo state, Brazil) the identified chemotype was characterized by large quantities of α-pinene, γ-terpinene and *p*-cymene [66,67]. The main components in the EOs from plants found in the Taiwan chemotype [77] were 1,8-cineole, α-terpinyl acetate and α-pinene, while the same chemotypes cultivated in Argentina [130] showed the presence of 52.7% of α-pinene, 18.4% of 1,8-cineole and 8.7% of *p*-cymene. Concerning EO extraction yields, species cultivated in Botucatu and in Argentina are low yielding (0.31% and 0.36%, respectively) while good extraction yields were observed for plants from Goiás state (Brazil) and Taiwan (2.0% and 3.01%, respectively).

In Brazil *E. grandis* is widely cultivated and used for cellulose pulp and paper production. Since its leaves have a high EO content (2.0%), further investigation to evaluate the use of such an industrial residue for EO production could constitute in a good business opportunity for the companies involved.

### 2.6. Eucalyptus saligna Smith

The species *E. saligna* is widely cultivated in Brazil for cellulose pulp production and is constituted of several chemotypes, some of them rich in 1,8-cineole. Another example of the 1,8-cineole chemotype is found in plants cultivated in the Democratic Republic of the Congo [53] which EO contained 61.3% of this component. Two studies conducted in Argentina [63,64,82] found the same chemotypes producing EOs with 1,8-cineole contents equal to 93.2% and 34.0%, respectively. Several studies carried out in Brazil, in different states, have revealed different EO compositions of *E. saligna*. A 1,8-cineole chemotype was found in plants cultivated in Rio Grande do Sul state [116]. Chemotypes presenting α-pinene as major component were found in plants cultivated in Minas Gerais state [68], which presented 92.3% of this compound, and in São Paulo state [66,67,132]. Finally, species cultivated in Nigeria [73] constituted a chemotype rich in α-thujene. In all the aforementioned studies of *E. saligna* EOs, the best extraction yields (1.42%) were obtained in the state of Minas Gerais [68].

### 2.7. Eucalyptus tereticornis Smith

The yields of EOs from *E. tereticornis* cultivated in different places varied from 0.45% to 2.3%. Two studies conducted in Benin found EOs presenting *p*-cymene as the main component [89,134]. The EOs of *E. tereticornis* cultivated in the Democratic Republic of the Congo also revealed *p*-cymene as the major component [53]. Lucia et al. [63,64] reported that *E. tereticornis* EOs are rich in β-phellandrene, 1,8-cineole and *p*-cymene. Toloza and co-workers [82] examined EOs containing 1,8-cineole, *p*-cymene, and γ-terpinene as the major components. A recent work by Filomeno and co-workers [68] reported that *E. tereticornis*, cultivated in Minas Gerais state in Brazil, produces high quantities (2.3%) of EOs rich in β-pinene, 1,8-cineole and α-pinene. This plant has a potential to be commercially explored as a source of EOs.

Based on the data described above, a large chemical variability is observed among *Eucalyptus* EO species. Such variation can be attributed to several factors including climate, soil type, plant age, nature (wet or dried) of the material used in the extraction, vegetative cycle stage, and time of the day when harvesting is done [35,95,99,138,139,140,141].

Since the chemical composition of the *Eucalyptus* EOs is directly associated with their biological activities, the following discussion will be focused on such activities and on the multiple applications of such EOs.

## 3. Biological Activities of *Eucalyptus* EOs

Several studies on antioxidant and antimicrobial activities of EOs from eucalyptus have been published in recent years [14,15,17,21,49,142,143,144,145,146,147,148]. Significant insecticide, antibacterial and fungicide effects have also been observed for EOs produced by *Eucalyptus* species [53,63,66,75,106]. Antimicrobial, acaricidal, insecticidal and herbicidal activities associated with EOs from the leaves of *Eucalyptus* are reported in several articles each year, demonstrating the importance of this research field. Such bioactivities are highly dependent on the EOs chemical composition, as discussed and illustrated in the following discussion.

### 3.1. Antimicrobial Activity

*Eucalyptus* EOs were evaluated against several Gram-positive and Gram-negative bacterial strains (Table 2) as well as against various fungal species (Table 3). The EOs showed different degrees of efficiency against the evaluated species. Among the bacterial strains, the pathogenic Gram-positive *Staphylococcus aureus* was most sensitive to EOs obtained from several *Eucalyptus* species. From the data available, *Pseudomonas aeruginosa* corresponded to the most resistant bacterial species. The yeast species *Candida albicans* also exhibited high sensitivity to the EOs.

#### 3.1.1. Antibacterial Activity

The EOs from *E. staigeriana* presented high antimicrobial activity against all evaluated microorganisms (Table 2). By using the agar diffusion method, *E. staigeriana* EOs presented the highest activity against *S. aureus* with inhibition zone diameter (izd) superior to 90 mm (the growth of the microorganism was inhibited over the entire Petri dish). This value was four times superior to the inhibition zone diameter caused by chloramphenicol, the commercial antibiotic used as positive control in the biological assays [17]. In the same investigation, it was demonstrated that *E. dives* EOs were also very effective against *S. aureus* (izd 52.3 mm in diameter, a value approximately two times higher than the izd observed for chloramphenicol).

Derwich et al. [127] have demonstrated the efficiency of *E. globulus* EOs against Gram-negative *E. coli* and Gram-positive *S. aureus* and *S. intermedius*. These authors found that *E. globulus* EOs presented excellent activity on *E. coli* in the agar disc diffusion assay (izd = 48.15 mm) compared to *S. aureus* (izd = 13.5 mm) and *S. intermedius* (izd = 10.26). The minimum inhibitory concentration (MIC) for *E. coli* corresponded to 0.15 mg/mL while for *S. aureus* and *S. intermedius* the values corresponded to 0.75 mg/mL and 1.08 mg/mL, respectively.

The effects of *E. globulus* EOs on 14 food spoilage microorganisms have been investigated using liquid and vapour fase agar dilution/well diffusion method and disc volatilization method [43]. The MIC found from such methods varied in the range of 2.25–9.0 mg/mL for bacterial and fungal strains. It was observed that MIC obtained for Gram-positive *B. subtilis* and *S. aureus* were lower than MIC values found for Gram-negative *E. coli*, *P. aeruginosa*, and *P. fluorescens* [43]. In general, significantly higher antimicrobial activities were observed in the vapour phase. As previously mentioned, 1,8-cineole is the main component of *E. globulus* EOs. It has been demonstrated that this compound has antimicrobial activity against several microorganisms including *S. aureus* [149], *E. coli* and *B. subtilis* [150,151].

Vratnica and co-workers [126] investigated the antimicrobial effects of *E. globulus* EOs against 17 microorganisms, including food poisoning and spoilage bacteria and human pathogens. In general, the EOs were highly active against the evaluated microorganisms. The agar disc diffusion method was utilized and filter paper discs were impregnated with *E. globulus* EOs (5, 10, 15, 20 and 30 μL). In these assays, the highest inhibition zone diameter (izd) values were observed for *S. pyogenes* (25–51 mm), *S. aureus* (22–48 mm), and *E. coli* (23–47 mm). The broth microdilution method was used to determine MIC which ranged from 0.09 mg/mL to 3.13 mg/mL. The highest MIC values were found for *P. aeruginosa* and *Salmonella infantis* (3.13 mg/mL) and the lowest for *S. aureus*, *E. coli* and *S. pyogenes* (0.09 mg/mL). In this work, no information about the compounds possibly responsible for the biological activity was provided.

The EOs from *E. camaldulensis* were tested against a panel of 12 bacteria strains, and the most sensitive microorganism was *B. subtilis*. For this microorganism, the EOs caused izds in the range of 19.3 mm to 29.3 mm at different volumes (20, 30, 40, 50 and 100 μL) in the agar diffusion method. When tested against *L. monocytogenes* and *S. aureus*, the EOs caused significant growth inhibition of the microorganisms, as attested by the corresponding izds ranging from 14.6 to 25.0 mm [70].

The biological assays conducted with EOs of *E. odorata* displayed the best results against *S. aureus* (izd = 27.4 mm) as determined in the agar diffusion method, followed by *S. agalactiae* (izd = 19.4 mm), *H. influenzae* (izd = 19.2 mm), *S. pyogenes* (izd = 19.0 mm) and *S. pneumoniae* (izd = 17.4 mm). Moreover, *E. maidenii* exhibited good activity against *S. aureus* (izd = 22.8 mm) [87,152].

Antimicrobial activities of *Eucalyptus* spp. EOs against resistant bacterial strains have also been described. For instance, *P. aeruginosa* is known for its high intrinsic resistance against antibiotics. This fact has been attributed to the very restrictive outer membrane barrier of the bacteria, being highly resistant even to synthetic drugs [17,49]. The EOs of *E. camaldulensis* and *E. tereticornis* exhibited relevant activity against *P. aeruginosa* (izd ~16.0 mm) [53]. The EOs from *E. cinerea* were less active (izd = 7.0 mm) when tested against *P. aeruginosa* [84].

In general, Gram-positive bacterial strains are more sensitive to *Eucalyptus* EOs than the Gram-negative ones [17,43,84]. This can be rationalized considering that Gram-negative bacteria possess a lipopolysaccharide membrane which is restrictive to the diffusion of hydrophobic compounds. In addition, the direct contact between the hydrophobic components of the EOs and the phospholipid bilayer of the cell membrane can occur in Gram-positive bacteria. As a consequence, the components exert their effects such as increase in the permeability to ions, leakage of vital intracellular components or compromise bacterial enzymes [43,84].

#### 3.1.2. Antifungal Activity

*Eucalyptus* EOs also cause growth inhibition of some fungal species (Table 3), as in the case of *C. albicans*. Vratnica and co-workers [126] reported that *E. globulus* EOs were two times more effective (izd = 14–46 mm) than nystatin, a drug used to treat fungal infections on the skin, mouth, vagina, and intestinal tract. The authors attributed this effect to the high content of 1,8-cineole in *E. globulus* EOs (85.8%). This information should be taken with caution since in another study the correlation between 1,8-cineole content and antifungal activity was not confirmed [77]. Gilles and co-workers [17] reported the effect of *E. staigeriana* (izd = 26.7 mm), *E. dives* (izd = 15.4 mm) and *E. olida* (izd = 12.6 mm) EOs on *C. albicans*. In this study, the superior antifungal activity of *E. staigeriana* EOs was attributed to the presence of 1,8-cineole (34.8%). Low activity against *C. albicans* was observed for EOs extracted from *E. robusta* and *E. saligna*, both without 1,8-cineole [132]. It should be noted that in the above cited studies no bioassays were conducted with pure 1,8-cineole, which could evaluate if 1,8-cineole has synergistic or antagonistic effect with other components of the EOs.

Tyagi and Malik [43] investigated the effect of EOs from *E. globulus* on several fungal species and reported MIC values ranging from 2.25 to 9 mg/mL. The superior limit value was observed for *P. digitatum* and *A. niger*. For *A. flavus*, *R. nigricans* and *F. oxysporum* a MIC of 4.5 mg/mL was found, while for *Mucor* spp. and *C. albicans* MIC of 2.25 mg/mL was reported.

In a recent study, it has been found that EOs from *E. erythrocorys* significantly reduced the growth of fungal species *B. sorokiniana* (79.6%) and *B. cinerea* (78.5%) [158].

The evaluation of antifungal activity of *E. citriodora* EOs, in concentration of 10 mg/disc, revealed that these EOs completely inhibit the growth of *C. cladosporioide*, *M. verrucaria* and *T. viride*. On the contrary, the growth of *A. clavatus*, *A. niger* and *P. citrinum* were partially inhibited (90.7%, 54.6% and 86.0%, respectively). Such antifungal activities were ascribed to the main components of *E. citriodora* EOs, namely citronellal (49.5%) and citronellol (11.9%) [77].

Lipid peroxidation and microbial contamination are two problems related to deterioration of food, an important issue for the food industry [39]. The addition of antioxidants is a well known strategy used to retard or even stop oxidation processes in food. Due to the carcinogenicity associated with some synthetic antioxidants, their use is restricted. In this context, an increased interest in the use of natural additives to control food oxidation has been observed. The use of EOs has been considered by the food industries as an alternative to overcome food deterioration [161,162]. Natural products presenting antioxidant activity has also been taken into consideration since some compounds with antioxidant activity can also be utilized as antimicrobials [37,163].

Infections caused by fungi and bacteria represent an important issue due to development of species resistant to well known fungicides and antibiotics [164]. Considering the relevant information available in the literature concerning the antimicrobial activity of *Eucalyptus* EOs, the employment of such can also be considered a viable alternative to overcome the resistance problem.

Synthetic fungicides are typically employed to prevent the contamination of food commodities from fungal deterioration as weel as from mycotoxin contaminations. However, the use of such substances is not free from side effects, as residual toxicity that contributes to the development of fungal resistance. This is particularly true when the fungi are exposed to fungicide sub-lethal concentrations. The use of EOs has been considered as an alternative to overcome the reported problems associated with synthetic fungicides and protection of food commodities [159,165]. Althought a promising strategy, further investigation in this area is still required to achieve a commercial product.

### 3.2. Acaricidal Activity

An acaricide can be defined as any substance or mixture of substances intended to prevent, destroy, repel, or mitigate ticks and mites. A number of studies have demonstrated the acaricidal effects of EOs obtained from different species of *Eucalyptus* (Table 4).

The effects of EOs from *E. citriodora*, *E. globulus* and *E. staigeriana* on the tick species *B. microplus* were evaluated at several doses (1%, 5%, 10%, 20% and 30% in methanol). The EOs from *E. citriodora* and *E. staigeriana* were the most active, causing 100% mortality of the larvae at 10% concentration. To achieve the same 100% mortality, it was required 20% of the EOs of *E. globulus* [91].

The EOs from *E. citriodora* are also toxic to the mite species *T. urticae* and *N. californicus*. A mortality bioassay was used to determine the LD_50_ of EOs (LD stands for lethal dose; LD_50_ denotes the dose likely to cause death in 50% of mites). The determined LD_50_ values were 19.3 μg/cm^3^ for *T. urticae* and 21.4 μg/cm^3^ for *N. californicus* [100].

Acaricidal effects were observed for EOs of *E. approximans*, *E. bicostata*, *E. maidenii* and *E. sideroxylon* on *T. urticae* females. At the concentrations of 0.5% and 1.0%, the reported observed mortalities were as follows: *E. approximans* (67% at 0.5%; 83.1% at 1.0%), *E. bicostata* (67.8% at 0.5% and 82.5% at 1.0%), *E. maidenii* (82.2% at 0.5% and 100.0% at 1.0%), *E. sideroxylon* (78.8% at 0.5% and 79.4% at 1.0%) [166].

The contact toxicity assay was used to evaluate the effects of *E. citriodora* EOs on the mite species *D. gallinae*. Using a dose of 0.21 mg/cm^2^ and after 24 h of exposure, 85% mortality was observed [102]. The effect of *E. citriodora* EOs was tested on larvae of the mite species *Amblyomma cajennense* and *Anocentor nitens.* In the biological evaluation, the concentrations ranged from 6.25% to 50%. For *A. cajennense*, the acaricidal effect varied from 10.8% to 53.1% mortality; for *A. nitens*, a more sentitive species, the mortality ranged from 20.1% to 100% [167].

The acaricidal activity of EOs from *E. camaldulensis* on *V. destructor* mite was also investigated and a LD_50_ of 1.74 μL/L of air was found [71].

From the surveyed literature, it was clear that the acaricidal effects of EOs from eucalyptus in some cases are high and could lead to the development of an environmental friendly commercial products to control such parasites. However, the works reported are limited to nine species of eucalyptus, concentrated in five countries. Therefore, considering the large disponibility and diversity in chemical composition of EOs from eucalyptus, we believe that EOs endowed with more potent and specific acaricidal activities are still to be discovered and converted into commercial products.

### 3.3. Insecticidal Activity

There are more than 1,000,000 reported species of insects, with approximately 10,000 of them showing crop-eating behavior; of these, approximately 700 species cause the majority of global pest-related damage to crops. Moreover, several diseases that affect man are transmitted by insects [168]. Therefore, controlling insects is highly desirable and necessary to improve human quality of life and health. Compounds obtained from natural sources have been investigated for their insecticidal activities [169,170,171]. Many such compounds have been used as models for the development of active ingredients to control insects [172,173,174,175,176,177,178,179,180,181,182,183,184,185]. In this regard, EOs have attracted the attention of researchers as an alternative to synthetic chemical-based insect control [186,187,188,189,190,191,192,193]. As shown in Table 5, EOs from many *Eucalyptus* species show positive results in controlling a variety of insect species.

The insecticidal activity of EOs from *E. globulus* was evaluated against the larvae and pupae stages of house fly *M. domestica* (Diptera: Muscidae). The effects of the EOs were assessed via fumigation and contact bioassays. Considering the larvae stage, in the contact assay the observed lethal concentration (LC_50_) ranged from 2.73 to 0.60 μL/cm^2^ for different days of observation, while the 50% lethality time (LT_50_) varied from 1.7 to 6 days. The observed LC_50_ values in the fumigation test were 66.1 and 50.1 μL/L after 24 and 48 h, respectively. Pupicidal activity was reported in terms of inhibition percentage rate (IPR) which was 36.0% to 93.0% for contact assay and 67.9% to 100% for fumigation test [122]. In another investigation, the EOs of *E.* cinerea were evaluated against adult stage of *M. domestica via* fumigation assays. An LC_50_ of 5.5 mg/dm^3^ was found and the mortality of the insects was observed in a period of time of less than 15 min [81]. The major component in the oil used in this work was 1,8-cineole (56.9%), a component of several other EOs with insecticidal activity.

The effects of EOs from *E. gunnii*, *E. tereticornis*, *E. grandis*, *E. camaldulensis*, *E. dunnii*, *E. cinerea*, *E. saligna*, *E. sideroxylon*, *E. globulus* ssp. *globulus*, *E. globulus* ssp. *maidenii*, *E. viminalis* and the hybrids *E. grandis* x *E. tereticornis and E. grandis x E. camaldulensis* were tested on *A. aegypti* larvae. The best results were observed for *E. dunnii*, *E. gunnii*, *E. tereticornis*, *E. camaldulensis* and *E. saligna* which presented, respectively, LC_50_s of 25.2, 21.1, 22.1, 26.8 and 22.2 mg/L. In this work, a correlation between the toxicity effect and the EOs contents of 1,8-cineole and *p*-cymene was found. However, other *Eucalyptus* species producing EOs with high content of 1,8-cineole and low concentration of *p*-cymene (*E. cinerea*, *E. globulus* ssp. *maidenii*, *E. globulus* ssp. *globulus*, *E. sideroxylon*, *E. viminalis*, *E. grandis, E. tereticornis*, *E. grandis*, and *E. camaldulensis*) had a lower effect on *A. aegypti* (larval mortality < 50% after 24 h at 40 ppm) [63,64].The vapor of the EOs of the aforementioned *Eucalyptus* species were also tested on *A. aegypti* adults. The toxicity was determined as the number of knockdown mosquitoes as a function of time. The fumigation toxicity was expressed as knockdown effect time (KT_50_) which varied from 4.2 to 12.0 min. The best result was observed for *E. viminalis* EOs. In this case, a direct correlation was found between the EO 1,8-cineole contents and toxicity level [64].

The investigation carried out by Cheng and co-workers [78] demonstrated larvicidal activity of *E. camaldulensis* and *E. urophylla* EOs against *A. aegypti* and *A. albopictus*. The EOs from *E. camaldulensis* presented the best results with LC_50_ of 31.0 and 55.3 μg/mL, respectively (the corresponding LC_90_ were 71.8 and 192.4 μg/mL for *A. aegypti* and *A. albopictus*, respectively). The larvicidal activity of individual components of *E. camaldulensis* EOs was also assessed. It was observed that α-terpinene caused the highest larvicidal activity (LC_50_ of 14.7 μg/mL and LC_90_ of 39.3 μg/mL for *A. aegypti*; LC_50_ of 25.2 μg/mL and LC_90_ > 50.0 μg/mL for *A. albopictus*). The EOs from *E. citriodora* were toxic to third and fourth instar of *A. aegypti* (LC_50_ 71.2 ppm) [103].

*L. longipalpis* is the vector of *Leishmania chagasi*, a protozoan species which is responsible for 90% of visceral leishmaniasis in Brazil. The effects of EOs of *E. staigeriana, E. citriodora and E. globulus* were evaluated on eggs, larva and adult phases of *L. longipalpis*. All EOs were active on the evaluated phases being *E. staigeriana* the most effective one, followed by *E. citriodora* and *E. globulus* [95]. Although the authors have not assessed individual essential oil components for their activities, it is worth pointing out that the EOs had citronellal as major component (71.8%), a compound known for its insecticidal activity.

The major pest of maize *S. zeamais* is known to attack both standing crop and the stored cereal. Investigations on the insecticidal and repellent effects of *E. dunnii, E. saligna*, *E. benthamii*, *E. globulus* and *E. viminalis* EOs on *S. zeamais* were carried out. By using the contact cytotoxicity assay on filter paper, EOs from *E. globulus* and *E. viminalis* caused 100% mortality at concentrations of 0.16 and 0.23 μL/cm^2^ after 24 h of exposure, respectively. Considering this parameter, the concentration values for other EOs were as follows: 0.42 μL/cm^2^ for *E. dunnii*, 0.65 μL/cm^2^ for *E. saligna* and 2.60 μL/cm^2^ *E. benthamii*. A regression analysis allowed the calculation of LC_50_ values: *E. viminalis* (0.08 μL/cm^2^); *E. globulus* (0.10 μL/cm^2^); *E. dunnii* (0.16 μL/cm^2^); *E. saligna* (0.25 μL/cm^2^) and *E. benthamii* (0.79 μL/cm^2^). The analysis of essential oil content and mortality activity resulted in a correlation between 1,8-cineole content and LC_50_. Thus, it is plausible to consider this compound responsible for the observed activity. Using the calculated LC_50_, it was possible to determine the repellency activity for all *Eucalyptus* EOs [116].

Among the components of EOs, monoterpenoids have contributed to fumigant activity against storage product pests [199], and it has been shown that they are lethal to insects by inhibiting the enzyme acetylcholinesterase activity (AChE) [200]. The repellent activity of *E. saligna*, *E. camaldulensis*, *E. globulus* and *E. citriodora* EOs were also assayed against *S. zeamais*. Y-shape olphatometer bioassay was utilized and the concentration tested range from 0.002 to 2 μL/μL. EOs were dissolved in hexane and at the highest concentration, *E. camaldulensis* and *E. citriodora* EOs presented the best repellent activity (74.35% and 69.15%, respectively), followed by *E. globulus* (53.68%) and *E. saligna* (40.5%). The repellent activity observed for *E. camaldulensis* EOs was higher than that observed for the positive control *N*-*N*-diethyl *m*-toluamide (DEET)*.* Some individual constituents of the EOs were assayed and the highest repellent activity was associated with 1,8-cineole content (70.87%) [72].

The fumigant toxicity of several EOs was evaluated on *S. oryzae* (also known as the rice weevil). The best activity was associated with *E. globulus* EOs (LD_50_ of 28.9 μL/L of air). Individual assessment of 1,8-cineole, the major component of *E. globulus* EOs, revealed a LD_50_ of 23.5 μL/L of air for the fumigant toxicity [113].

The EOs from *E. globulus*, rich in 1,8-cineole, had their antitermite activity evaluated against *O. assamensis*. At the concentration of 2.5 mg/g, *E. globulus* EOs caused 80% mortality while 70% was observed for pure 1,8-cineole [120]. These results suggest that other compounds present in the oil might be enhancing the effect of 1,8-cineole.

*P. humanus capitis* (head louse) is an obligate ectoparasite responsible for the head lice infestation, also known as pediculosis capitis, nits or cooties. Several reports have described the effects of *Eucalyptus* EOs on *P. humanus capitis*. The fumigant toxicity assay was utilized to evaluate the effect of EOs from *E. sideroxylon*, *E. globulus* ssp. *globulus*, *E. globulus* ssp. *maidenii*, *E. dunnii*, *and E. gunnii* on head lice resistant to permetrin. Among the evaluated EOs, the most efficient ones were *E. sideroxylon*, *E. globulus* ssp. *globulus* and *E. globulus* ssp. *maidenii* presenting, respectively, KT_50_ of 24.75, 27.73, and 31.39 min [110]. A similar investigation conducted with EOs from *E. cinerea*, *E. viminalis* and *E. saligna* revealed KT_50_ values of 12.0, 14.9, and 17.4 min [82]. A comparative investigation on the effect of EOs from hybrids (*E. grandis* x *E. camaldulensis* and *E. grandis* x *E. tereticornis*) and no-hybrids (*E. grandis*, *E. camaldulensis*, and *E. tereticornis*) eucalyptus species on *P. humanus capitis* was carried out. The fumigant activity of hybrids was higher than non-hybrid ones. The observed KT_50_ values for the hybrid were *E. grandis* x *E. tereticornis* (12.99 min) and *E. grandis* x *E. camaldulensis* (13.63 min). For the non-hybrid, the values for KT_50_ parameter were *E. grandis* (25.57 min), *E. camaldulensis* (35.01 min) and *E. tereticornis* (31.31 min) [65].

*E. citriodora* leaves has been traditionally used as insecticide repellent, especially by low income families to protect themselves against mosquitoes [201].

The red flour beetle *T. castaneum* is a worldwide pest of stored products, particularly food grains. The EOs of *E. citriodora*, rich in citronellal, citronellol and isopulegol, presents repellent activity against this beetle species (0.084 mL/L dose repellent media after 4 h of exposure). The observed activity was higher than the commercial product ethyl 3-(*N*-acetyl-*N*-butylamino) propionate used as positive control [104].

The evaluation of fumigant activity of EOs from *E. camaldulensis*, *E. astringens*, *E. leucoxylon*, *E. lehmannii* and *E. rudis* against the pests of stored products *E. kuehniella*, *E. cautella* and *E. ceratoniae* showed that *E. camaldulensis* EOs present high toxicity on *E. cautella* and *E. kuehniella* (LC_50_ = 11.07 and 26.73 μL/L of air, respectively). Considering *E. ceratoniae*, the most effective EOs were extracted from *E. rudis* (LC_50_ = 31.4 μL/L of air) [79]. In another study, the effects of *E. camaldulensis* and *E. leucoxylon* EOs on larvae and adult stages of *E. ceratoniae* were investigated. The EOs presented bioactivity on both stages of the insect development. For adult stage, 100% mortality was achieved for both EOs after 120 h of exposure at 26.31 μL/L of air; at higher concentration (131.58 μL/L of air) the exposure time was reduced to 48 h. The LC_50_ after 24 h of exposure corresponded to 12.07 μL/L of air and 21.75 μL/L of air for *E. camaldulensis* and *E. leucoxylon*, respectively. Considering the larvae stage, 100% mortality was observed at 131.58 μL/L of air after 264 h of exposure [80].

The EOs from *E. tereticornis*, at the concentration of 160 ppm, caused 100% mortality on the larves of *Anopheles stephensi* [202]. The observed insecticidal activity of *E. tereticornis* EOs on *A. gambiae* was associated to *p*-cymene and 1,8-cineole as demonstrated by the biological assays conducted with these individual components [89].

### 3.4. Herbicidal Activity

Weeds compete with crops for water, nutrients and light, and controlling their growth is of fundamental importance in modern agriculture. It is estimated that approximately 10% of all plant species are weeds, corresponding to approximately 30,000 species. Among them, some 1800 cause serious economic losses in crop production [203].

The observation of plant growth regulation effects caused by EOs has attracted the attention of researchers toward the possibility of utilizing these natural sources for weed control [136,204]. Such investigations are important from the viewpoint of evolution of resistance of weeds to traditional herbicides. There is a constant need for the development of weed control agents that are environmentally benign, present low toxicity to mammals, less recalcitrant, and can be applied in less quantity [205,206,207]. In this regard, nature has been considered an important source of compounds that can be explored to provide herbicides that can meet the aforementioned criteria [206,208,209].

As shown in Table 6, several studies have been conducted on the phytotoxic effects of *Eucalyptus* EOs on weeds [31,136,210,211]. It has been demonstrated that these EOs inhibit and/or retard the germination of seeds. Effects on crop species have also been described [18].

The phytotoxic effect of *E. citriodora* EOs collected from leaves at differents stages (juvenile and adult leaves) and fallen (senescent leaves and brown leaf litter) has been investigated on two weed species (*E. crus-galli* and *A. viridis*) and two crops (*T. aestivum* and *O. sativa*). As a general trend, the adult leaf EOs presented superior phytotoxicity compared to leaf litter EOs. In a subsequent investigation, Batish and co-workers [26] examined the phytotoxic effects of EOs extracted from decaying leaves of *E. citriodora* against weed species *C. occidentalis* and *E. crus-galli*. Also, the phytotoxicity of EOs major components, i.e., citronellal and citronellol, were also assessed. The EOs exhibited superior effect on the germination of *C. occidentalis* (I_50_ = 1.09 mg/mL) compared to *E. crus-galli* (I_50_ = 1.49 mg/mL). The EOs presented similar effects on root elongation (I_50_ = 0.31 mg/mL for *C. occidentalis* and 0.35 mg/mL for *E. grus-galli*). Comparing the effect of the major components on germination, citronellal was more effective in inhibiting the germination (I_50_ = 0.55 mg/mL and 0.14 mg/mL for *C. occidentalis* and *E. grus-galli*, respectively). On the contrary, citronellol caused a more pronounced effect on root elongation (I_50_ = 0.13 mg/mL and 0.09 mg/mL for *C. occidentalis* and *E. grus-galli*, respectively).

Silverleaf nightshade (*S. elaeagnifolium*) is a perennial and agressive weed species common in Australia. The effect of five selected *Eucalyptus* EOs from Australia, namely *E. brockwayii*, *E. dundasii*, *E. melliodora*, *E. salubris* and *E. spathulata*, on germination and root elongation of *S. elaeagnifolium* was evaluated. The EOs from *E. salubris* caused the highest (73%) inhibitory effect on germination. This effect was superior to that observed by commercial eucalyptus EOs (38% of inhibition index) purchased from the market and used as positive control. In terms of root growth inhibition, *E. salubris* was again the most effective EOs (reduction of 84% of root elongation when applied at 10 μL/dish). At the same dose, commercial eucalyptus EOs caused only 41% decrease in root length [212]. The phytotoxic effects of aqueous volatile fractions of the aforementioned EOs, i.e., the water soluble volatile fractions obtained along with the EOs (water insoluble fractions) during the steam distillation process were also assessed. It was also observed strong phytotoxic effects on germination, shoot length and root elongation of *S. elaeagnifolium* [213].

Shingh and co-workers [31] investigated the herbicidal effect of EOs produced by *E. citriodora* against the weed species *Parthenium hysterophorus*. They found that germination has been fully inhibited by the EOs (dose used 5.0 nL/mL). Plants of *P. hysterophorus* (4-week-old) were sprayed with different concentrations of EOs (0–100 μL/mL). A week after spraying, damage and decreased chlorophyll content and respiratory activity as the EOs concentration increased was noticed. When sprayed with concentrations up to 50 μL/mL, plants showed recovery over time. However, when the weed species were sprayed with 75 μL/mL and 100 μL/mL, plants died after two weeks. Moreover, plants sprayed with 50 μL/mL and concentrations higher than that were dessicated and wilted. *E. citriodora* EOs caused rapid electrolyte leakage at concentrations of 5–75 μL/mL indicating an effect on membrane integrity.

Phytotoxic effects of *E. citriodora* EOs on the crops *S. bicolor* L. (sorghum) and *C. sativus* L. (cucumber) have been reported. From the biological essays, an allelopathic effect was observed mainly causing germination and radicule growth inhibition of *S. bicolor* and *C. sativus* seeds. It was also observed that the increase of EOs concentration (0 to 5000 ppm) leads to a linear decrease in the germination as well as in the radicule length of *S. bicolor* [99].

## 4. Concluding Remarks

The world production and trade of EOs from several *Eucalytus* species is dominated by China which is the biggest producer of EOs rich in 1,8-cineole [215]. Other important producers include South Africa, Portugal, Spain, Brazil, Australia, Chile and Swaziland [45]. There are important aspects to be considered with respect to cultivation of *Eucalyptus* spp. aimed for production of EOs such as environmental, genetic variation and leaf type.

The majority of EOs produced by *Eucalyptus* are rich in monoterpenes. For medicinal purposes, the value of *Eucalyptus* EOs is directely associated to its content of 1,8-cineole that should be at least 70% in mass. It should be mentioned that medicinal EOs are designated in terms of 1,8-cineole content. Typical descriptions for such oils are: “*Eucalyptus* oil China 80%”, “*Eucalyptus* oil 70/75% Spain/Portugal” and “*Eucalyptus* oil 80/85% Spain/Portugal”. The highest price is associated with an essential oil know as ‘eucalyptol’ which contains about 98% 1,8-cineole [45,216]. In Table 7 the main *Eucalyptus* species that have been used for the extraction of medicinal essential oils are listed [217,218].

In several cases according to the source, after extraction certain crude EOs have to be rectified to increase the percentage of 1,8-cineole required for medicinal purposes.

The EOs intended for use in perfumery are rich in citronellal, citronellol and geranyl acetate. One important source of perfumery *Eucalyptus* EOs is *E. citriodora* in which the major component is citronellal and its content should be in the range 65%–85%. The essential oils of *E. citriodora* are used in whole form for fragrance purposes, usually in lower-cost soaps, perfumes and disinfectants, but their main use is as a source of citronellal for the chemical industry [45,216,219].

The term industrial oil is commonly used to describe the use of the EOs as raw materials for the isolation of their chemical constituents. The industrial EOs are characterized by high levels of phellandrene and piperitone, and mainly obtained from *E. dives* species [216].

As described by Coppen [220] “any attempt to accurately quantify and analyse production and consumption trends for *Eucalyptus* oil is fraught with difficulties. Unlike some other commodities, or some other EOs such as the citrus oils, quantitative information is not always available or accessible”.

Research on EOs is of fundamental importance considering the current applications of natural extracts and EOs in the food, cosmetic, perfume, pharmaceutical, and agrochemical industries. In this review, the large chemical variability that exists among EOs from several species of *Eucalyptus* was demonstrated. In addition, the usefulness of those EOs in terms of their antimicrobial, insecticidal, acaricidal, and phytotoxic activity was described. In some cases, the observed biological activity of the EOs is superior to that of the products available in the market, but there is very limited research about the mechanism of action of the biological activities of such EOs. Considering all such aspects, and taking into consideration that several species of *Eucalyptus* are still unexplored in terms of their essential oil content and composition, we envisage that investigations in this field will continue to be active in the future. New activities will be reported for *Eucalyptus* EOs and further details on their mechanisms of action will also appear in the future.

## Figures and Tables

**Table 1:** Some common chemical components of essential oils extracted from leaves of *Eucalyptus* spp. ^a^.

*Eucalyptus* spp.	Origin	Components of *Eucalyptus* EOs	EOs Yields (%)	Reference
*E. camaldulensis*	Argentina	1,8-cineole (19.1%), *p*-cymene (17.9%), β-phellandrene (16.3%)	0.38	[63,64,65]
	Brazil	1,8-cineole (52.8%), limonene (14.2%), γ-terpinene (6.8%), α-pinene (6.1%)	0.63	[66,67]
	Brazil	1,8-cineole (44.8%), α-phellandrene (22.9%), *p*-cymene (9.8%)	3.00	[68]
	Democratic Republic of the Congo	1,8-cineole (58.9%), myrtenol (4.3%), myrtenal (3.5%)	0.30 ^b^	[53]
	Egypt	1,8-cineole (60.3%), α-pinene (13.6%), γ-terpinene (8.8%)	-	[69]
	India	α-phellandrene (27.5%), β-pinene (23.5%), *m*-cymene (9.5%), 1,8-cineole (8.7%)	1.97 ^b^	[70]
	Iran	1,8-cineole (74.7%)	-	[71]
	Kenya	1,8-cineole (18.9%), α-cadinol (6.4%), β-phellandrene (2.6%)	-	[72]
	Nigeria	1,8-cineole (70.4%), β-pinene (9.0%), α-pinene (8.8%)	0.26	[73]
	Northern Cyprus	1,8-cineole (19.0%), β-caryophyllene (11.6%), carvacrol (9.1%)	-	[74]
	Pakistan	linalool (17.0%), 1,8-cineole (16.1%), *p*-cymene (12.2%)	1.90	[75]
	Spain	spathulenol (41.5%), *p*-cymene (21.9%)	0.71	[76]
	Taiwan	1,8-cineole (29.6%), limonene (15.2%), β-pinene (9.9%), α-pinene (9.7%)	3.48	[77]
	Taiwan	α-pinene (22.5%), *p*-cymene (21.7%), α-phellandrene (20.1%), 1,8-cineole (9.5%)	0.57	[78]
	Tunisia	1,8-cineole (20.6%), α-pinene (16.5%)	0.76–1.42	[79,80]
*E. cinerea*	Argentina	1,8-cineole (88.5%), α-terpineol (9.0%), α-pinene (2.0%)	-	[81]
	Argentina	1,8-cineole (79.8%), α-terpinyl acetate (8.2%)	2.48	[63,64]
	Argentina	1,8-cineole (62.1%), *p*-cymene (11.2%)	-	[82]
	Argentina	1,8-cineole (56.9%), α-pinene (6.4%)	-	[83]
	Brazil	1,8-cineole (83.6%), α-terpinyl acetate (5.4%), α-pinene (5.0%)	3.56–5.02	[84]
	Brazil	1,8-cineole (75.7%), α-terpineol (9.7%), α-pinene (6.2%)	6.07	[85]
	Tunisia	1,8-cineole (79.2%), α-terpinyl acetate (5.4%), α-pinene (4.1%)	3.00	[86]
	Tunisia	1,8-cineole (70.4%), α-terpineol (10.3%)	3.90	[87]
*E. citriodora*	Argentina	citronellal (76.0%), iso-isopulegol (9.0%), citronellyl acetate (7.3%)		[82]
	Australia	citronellal (68.9%), citronellol (7.6%), isopulegol (7.4%)	-	[88]
	Benin	citronellal (52.8%), citronellol (20.0%), citronellyl acetate (9.0%)	4.60	[89,90]
	Brazil	citronellal (94.9%), citronellyl acetate (2.6%), *trans* caryophyllene (2.5%)	-	[91]
	Brazil	citronellal (89.6%), citronellyl acetate (3.3%), 1,8-cineole (2.9%)	-	[92]
	Brazil	citronellal (82.3%), citronellyl acetate (7.8%), neothujan-3-ol (6.8%)	4.00	[93]
	Brazil	citronellal (76.0%), *neo-iso*-3-thujanol (11.8%)	0.66	[66,67]
	Brazil	β-citronellal (71.8%), (−)-isopulegol (7.3%), isopulegol (4.3%)	-	[94]
	Brazil	citronellal (71.8%), isopulegol (4.3%)	-	[95]
	Brazil	citronellal (71.1%), citronellol (8.8%)	-	[96]
	Brazil	citronellal (67.5%), citronellol (6.9%), menthol (6.1%)	-	[97]
	Brazil	citronellal (61.8%), isopulegol (15.5%), *β*-citronellol (7.9%)	-	[98]
	Brazil	citronellal (64.9%), *iso*-isopulegol (10.2%), citronellol (8.3%)	2.10	[99]
	China	citronellal (65.9%), citronellol (10.5%), 1,8-cineole (3.0%)	-	[100,101]
	China	citronellal (55.3%), citronellol (8.3%)	-	[102]
*E. citriodora*	Colombia	citronellal (49.3%), citronellol (13.0%), isopulegol (12.9%)	0.70	[103]
	Colombia	citronellal (40.0%), isopulegol (14.6%), citronellol (13.0%)	-	[104,105]
	Democratic Republic of the Congo	citronellal (72.7%), citronellol (6.3%), eugenol (3.5%)	1.63 ^b^	[53]
	India	citronellal (52.2%), citronellol (12.3%), isopulegol (11.9%)	0.60	[26]
	India	citronellal (48.3%), citronellol (21.9%), *iso*-isopulegol (12.7%)	2.36–4.80	[54]
	Indonesia	citronellal (90.1%), citronellol (4.3%)	-	[106]
	Kenya	1,8-cineole (11.2%), β-pinene (3.2%), terpinen-4-ol (3.1%)	-	[72]
	Pakistan	citronellal (22.3%), citronellol (20.0%)	1.82	[75]
	South Korea	citronellal (73.0%), isopulegol (6.7%)	-	[107]
	Taiwan	citronellal (49.5%), citronellol (11.9%), *iso*-isopulegol (10.4%)	1.89	[77]
	Tunisia	1,8-cineole (54.1%), α-pinene (23.6%)	3.30	[49,108]
*E. globulus*	Algeria	1,8-cineole (55.3%), spathulenol (7.4%), α-terpineol (5.5%)	2.53	[109]
	Argentina	1,8-cineole (77.9%), α-terpineol (6.0%)	2.25	[63,64]
	Argentina	1,8-cineole (76.7%), α-pinene (11.1%)	1.66	[63,110]
	Argentina	1,8-cineole (52.3%–62.1%)	1.31–1.49	[111]
	Australia	1,8-cineole (90.0%), α-pinene (2.2%)	-	[112]
	Australia	1,8-cineole (81.1%), limonene (7.6%), α-pinene (4.0%)	-	[113]
	Brazil	1,8-cineole (90.0%), tricyclene (3.0%)	-	[114]
	Brazil	1,8-cineole (85.8%), α-pinene (9.9%)	-	[91]
	Brazil	1,8-cineole (83.9%), limonene (8.2%), α-pinene (4.2%)	-	[95,115]
	Brazil	1,8-cineole (77.5%), α-pinene (14.2%)	3.10	[116]
	Democratic Republic of the Congo	1,8-cineole (44.3%), camphene (23.1%), α-pinene (9.3%), globulol (7.3%)	1.87 ^b^	[53]
	Egypt	1,8-cineole (21.4%), *o*-cimene (21.4%), α-pinene (6.7%), spathulenol (6.3%)	-	[117]
	Ethiopia	1,8-cineole (63.0%), α-pinene (16.1%)	-	[118]
	India	1,8-cineole (81.9%), limonene (6.6%)	-	[119]
	India	1,8-cineole (68.8%), α-pinene (2.8%)	-	[120]
	India	1,8-cineole (66.3%), *cis*-ocymene (21.3%), α-terpinyl acetate (3.4%)	-	[121]
	India	1,8-cineole (44.4%), limonene (17.8%), *p*-cymene (9.5%)	-	[43]
	India	1,8-cineole (33.6%), α-pinene (14.2%), limonene (10.1%)	-	[122]
	Indonesia	1,8-cineole (86.5%), α-pinene (4.7%)	-	[106]
	Iran	1,8-cineole (84.5%), limonene (8.50%)	-	[123]
	Iran	1,8-cineole (47.2%), spathulenol (18.1%), α-pinene (9.6%)	-	[124]
	Italy	1,8-cineole (84.9%), α-pinene (5.6%), *p*-cymene (5.3%)	-	[125]
	Kenya	1,8-cineole (17.2%), α-pinene (7.1%), spathulenol (6.5%)	-	[72]
	Montenegro	1,8-cineole (85.8%), α-pinene (7.2%), β-myrcene (1.5%)	1.80 ^b^	[126]
	Morocco	1,8-cineole (22.4%), limonene (7.0%), solanone (6.1%), β-pinene (5.2%)	1.21	[127]
	Pakistan	1,8-cineole (56.5%), limonene (28.0%)	1.89	[75]
	Spain	1,8-cineole (63.8%), α-pinene (16.1%)	-	[128]
*E. grandis*	Argentina	α-pinene (52.7%), 1,8-cineole (18.4%), *p*-cymene (8.7%)	0.36	[65,129,130]
	Brazil	*p*-cymene (59.6%), γ-terpinene (29.2%)	0.26	[68]
	Brazil	α-pinene (40.6%), γ-terpinene (16.3%), *p*-cymene (13.1%)	0.31	[66,67]
	Brazil	γ-terpinene (16.8%), *o*-cymene (16.7%), β-pinene (11.5%)	2.00	[93]
	Taiwan	1,8-cineole (19.8%), α-terpinyl acetate (12.8%), α-pinene (11.4%)	3.01	[77]
*E. saligna*	Argentina	1,8-cineole (93.2%)	-	[131]
	Argentina	1,8-cineole (93.2%), limonene (3.3%)	-	[82]
	Argentina	1,8-cineole (34.0%), *p*-cymene (21.3%), γ-terpinene (20.10%), α-pinene (13.0%)	0.36	[63,64]
	Brazil	1,8-cineole (45.2%), *p*-cymene (34.4%), α-pinene (12.8%)	0.50	[116]
	Brazil	*p*-cymene (25.6%), α-terpineol (9.3%), α-camphonellal (8.0%), 1,8-cineole (6.2%)	0.50	[93]
	Brazil	α-pinene (92.3%)	1.42	[68]
	Brazil	α-pinene (45.1%), *p*-cymene (22.5%), α-pinene oxide (11.3%)	0.40	[132]
	Brazil	α-pinene (25.9%), *p*-cymene (24.4%), γ-terpinene (24.6%)	0.19	[66,67]
	Democratic Republic of the Congo	1,8-cineole (61.3%), limonene (10.1%), *p*-cymene (7.2%)	0.78 ^b^	[53]
	Kenya	α-pinene (24.4%), 1,8-cineole (24.3%), *o*-cimene (9.9%), α-terpineol (8.8%)	0.38	[133]
	Nigeria	α-thujene (63.8%), 1,8-cineole (12.3%)	0.30	[73]
*E. tereticornis*	Argentina	1,8-cineole (37.5%), *p*-cymene (22.0%), γ-terpinene (10.8%)	-	[82]
	Argentina	β-phellandrene (22.6%), 1,8-cineole (18.6%), *p*-cymene (14.5%), α-phellandrene (9.4%)	0.60	[63,64,65]
	Benin	*p*-cymene (31.1%), β-phellandrene (9.7%)	-	[134]
	Benin	*p*-cymene (16.7%), caryophyllene oxide (14.2%), spathulenol (13.5%), cryptone (11.4%)	1.00	[89]
	Brazil	β-pinene (22.4), 1,8-cineole (19.3%), α-pinene (13.6%), α-phellandrene (10.3%)	2.30	[68]
	Democratic Republic of the Congo	*p*-cymene (28.6%), cryptone (17.8%), α-pinene (8.3%)	0.45 ^b^	[53]

^a^ The compounds are listed according to their decreasing quantities; ^b^ Fresh leaves; (-): not reported. A complete Table of common chemical components of essential oils extracted from leaves of *Eucalyptus* spp. is in Supplementary Materials.

**Table 2:** *Eucalyptus* spp. essential oils with antibacterial activities.

*Eucalyptus* spp.	Target Species	Reference
*E. alba*	*Bacillus subtilis*, *Citrobacter diversus*, *Escherichia coli*, *Klebsiella oxytoca*, *Klebsiella pneumoniae*, *Proteus vulgaris*, *Pseudomonas aeruginosa*, *Salmonella typhimurium*, *Staphylococcus aureus*	[53,73]
*E. astringens*	*Bacillus cereus*, *Escherichia coli*, *Listeria ivanovii*	[86]
*E. bicostata*	*Bacillus cereus*, *Listeria ivanovii*, *Staphylococcus aureus*, *Streptococcus pneumoniae*	[86,152]
*E. botryoides*	*Escherichia coli*, *Staphylococcus aureus*	[49]
*E. camaldulensis*	*Bacillus cereus*, *Bacillus subtilis*, *Citrobacter diversus*, *Escherichia coli*, *Klebsiella oxytoca*, *Klebsiella pneumoniae*, *Listeria monocytogenes*, *Proteus vulgaris*, *Pseudomonas aeruginosa*, *Shigella flexneri*, *Staphylococcus aureus*	[53,70,73,74,75]
*E. cinerea*	*Bacillus cereus*, *Escherichia coli*, *Listeria ivanovii*, *Pseudomonas aeruginosa*, *Staphylococcus aureus*, *Staphylococcus epidermidis*, *Streptococcus pyogenes*	[84,85,86,87]
*E. citriodora*	*Bacillus subtilis*, *Citrobacter diversus*, *Enterococcus faecalis*, *Escherichia coli*, *Klebsiella oxytoca*, *Klebsiella pneumoniae*, *Proteus vulgaris*, *Pseudomonas aeruginosa*, *Salmonella choleraesuis*, *Salmonella typhimurium*, *Shigella flexneri*, *Staphylococcus aureus*	[49,53,75,93,106]
*E. cloeziana*	*Escherichia coli*, *Staphylococcus aureus*	[93]
*E. crebra*	*Bacillus subtilis*, *Escherichia coli*, *Staphylococcus aureus*	[75]
*E. deglupta*	*Bacillus cereus*, *Bacillus subtilis*, *Escherichia coli*, *Klebsiella oxytoca*, *Klebsiella pneumoniae*, *Pseudomonas aeruginosa*, *Salmonella typhimurium*, *Shigella flexneri*, *Staphylococcus aureus*	[53,73]
*E. diversifolia*	*Enterococcus faecalis*, *Escherichia coli*, *Staphylococcus aureus*	[153]
*E. dives*	*Escherichia coli*, *Listeria monocytogenes*, *Pseudomonas fragi*, *Salmonella typhimurium*, *Staphylococcus aureus*	[17,154]
*E. globulus*	*Acinetobacter baumannii*, *Bacillus cereus*, *Bacillus subtilis*, *Citrobacter diversus*, *Enterococcus faecalis*, *Escherichia coli*, *Fusobacterium nucleatum*, *Klebsiella oxytoca*, *Klebsiella pneumoniae*, *Porphyromonas gingivalis*, *Pseudomonas aeruginosa*, *Pseudomonas fluorescens*, *Salmonella paratyphi*, *Salmonella typhi*, *Salmonella typhimurium*, *Shigella*, *Staphylococcus aureus*, *Staphylococcus intermedius*, *Staphylococcus sciuri*, *Staphylococcus warneri*, *Streptococcus pyogenes*	[43,53,75,106,109,118,123,124,125,126,127,128]
*E. gracilis*	*Klebsiella pneumoniae*, *Listeria monocytogenes*	[155]
*E. grandis*	*Escherichia coli*, *Salmonella choleraesuis*, *Staphylococcus aureus*	[93]
*E. lehmannii*	*Bacillus cereus*, *Escherichia coli*, *Pseudomonas aeruginosa*, *Staphylococcus aureus*	[86,152]
*E. leucoxylon*	*Bacillus cereus*, *Escherichia coli*	[86]
*E. maidenii*	*Bacillus cereus*, *Enterococcus faecalis*, *Escherichia coli*, *Listeria ivanovii*, *Staphylococcus aureus*	[86,152]
*E. melanophloia*	*Bacillus subtilis*, *Escherichia coli*, *Staphylococcus aureus*	[75]
*E. microcorys*	*Staphylococcus aureus*	[93]
*E. microtheca*	*Bacillus subtilis*, *Escherichia coli*, *Staphylococcus aureus*	[75]
*E. odorata*	*Enterococcus faecalis*, *Escherichia coli*, *Haemophilus influenzae*, *Streptococcus agalactiae*, *Staphylococcus aureus*, *Streptococcus pneumoniae*, *Streptococcus pyogenes*	[152]
*E. oleosa*	*Klebsiella pneumoniae*, *Listeria monocytogenes*	[155]
*E. radiata*	*Acinetobacter baumannii*, *Escherichia coli*, *Klebsiella pneumoniae*, *Pseudomonas aeruginosa*, *Salmonella typhimurium*	[128]
*E. robusta*	*Bacillus subtilis*, *Escherichia coli*, *Klebsiella oxytoca*, *Klebsiella pneumoniae*, *Pseudomonas aeruginosa*, *Salmonella typhimurium*, *Shigella flexneri*, *Staphylococcus aureus*	[53,132]
*E. saligna*	*Bacillus cereus*, *Bacillus subtilis*, *Citrobacter diversus*, *Escherichia coli*, *Klebsiella oxytoca*, *Klebsiella pneumoniae*, *Pseudomonas aeruginosa*, *Salmonella choleraesuis*, *Staphylococcus aureus*	[53,73,93,132]
*E. olida*	*Staphylococcus aureus*	[17]
*E. ovata*	*Escherichia coli*	[49]
*E. pellita*	*Bacillus subtilis*, *Escherichia coli*, *Pseudomonas aeruginosa*, *Staphylococcus aureus*	[156]
*E. platypus*	*Enterococcus faecalis*	[152]
*E. propinqua*	*Bacillus subtilis*, *Citrobacter diversus*, *Klebsiella oxytoca*, *Klebsiella pneumoniae*, *Salmonella typhimurium*, *Shigella flexneri*, *Staphylococcus aureus*	[53]
*E. radiata*	*Enterococcus faecalis*, *Escherichia coli*, *Klebsiella pneumoniae*, *Pseudomonas aeruginosa*, *Staphylococcus aureus*	[106]
*E. salmonophloia*	*Klebsiella pneumoniae*, *Listeria monocytogenes*	[155]
*E. salubris*	*Klebsiella pneumoniae*, *Listeria monocytogenes*	[155]
*E. sargentii*	*Bacillus subtilis*, *Escherichia coli*, *Klebsiella pneumoniae*, *Proteus vulgaris*, *Pseudomonas aeruginosa*, *Shigella dysenteriae*, *Staphylococcus aureus*, *Staphylococcus epidermidis*	[157]
*E. sideroxylon*	*Bacillus cereus*, *Listeria ivanovii*	[86]
*E. staigeriana*	*Enterococcus faecalis*, *Staphylococcus aureus*	[17]
*E. tereticornis*	*Bacillus subtilis*, *Citrobacter diversus*, *Corynebacteriaceae* spp., *Escherichia coli*, *Klebsiella oxytoca*, *Klebsiella pneumoniae*, *Proteus vulgaris*, *Pseudomonas aeruginosa*, *Shigella flexneri*	[53,134]
*E. urophylla*	*Bacillus subtilis*, *Escherichia coli*, *Klebsiella oxytoca*, *Klebsiella pneumoniae*	[53]

**Table 3:** *Eucalyptus* spp. essential oils with antifungal activities.

*Eucalyptus* spp.	Target Species	Reference
*E. astringens*	*Candida albicans*, *Microsporum canis*	[152]
*E. bicostata*	*Candida albicans*	[152]
*E. camaldulensis*	*Alternaria alternata*, *Aspergillus clavatus*, *Aspergillus niger*, *Candida albicans*, *Chaetomium globosum*, *Cladosporium cladosporioides*, *Lenzites sulphureus*, *Myrothecium verrucaria*, *Penicillium citrinum*, *Phanerochaete chrysosporium*, *Phaeolus schweintizii*, *Rhizopus solani*, *Trametes versicolor*, *Trichoderma viride*	[69,73,75,77]
*E. cinerea*	*Candida albicans*	[84,85]
*E. citriodora*	*Aspergillus clavatus*, *Aspergillus niger*, *Aspergillus* spp., *Botrytis cinerea*, *Chaetomium globosum*, *Cladosporium cladosporioides*, *Colletotrichum gloeosporioides*, *Colletotrichum musae*, *Cryphonectria parasitica*, *Fusarium oxysporum*, *Lenzites sulphureus*, *Myrothecium verrucaria*, *Penicillium citrinum*, *Phaeolus schweintizii*, *Phanerochaete chrysosporium*, *Phytophthora cactorum*, *Pyricularia grisea*, *Pythium ultimum*, *Rhizoctonia solani*, *Rhizopus solani*, *Trametes versicolor*, *Trichoderma viride*	[75,77,88,98,107]
*E. crebra*	*Aspergillus niger*, *Rhizopus solani*	[75]
*E. deglupta*	*Candida albicans*	[73]
*E. dives*	*Candida albicans*, *Saccharomyces cerevisiae*	[17]
*E. erythrocorys*	*Bipolaris sorokiniana*, *Botrytis cinerea*	[158]
*E. globulus*	*Aspergillus flavus*, *Aspergillus niger*, *Aspergillus parasiticus*, *Aspergillus* spp., *Candida albicans*, *Fusarium oxysporum*, *Mucor* spp., *Penicillium digitatum*, *Rhizopus nigricans*, *Rhizopus solani*, *Saccharomyces cerevisiae*, *Trichophyton* spp.	[43,75,114,118,126]
*E. gracilis*	*Aspergillus ochraceus*, *Candida albicans*, *Mucor ramamnianus*, *Saccharomyces cerevisiae*	[155]
*E. grandis*	*Aspergillus clavatus*, *Aspergillus niger*, *Chaetomium globosum*, *Cladosporium cladosporioides*, *Lenzites sulphureus*, *Myrothecium verrucaria*, *Penicillium citrinum*, *Phaeolus schweintizii*, *Phanerochaete chrysosporium*, *Trametes versicolor*, *Trichoderma viride*	[77]
*E. maidenii*	*Candida albicans*, *Trichophyton soudanense*	[152]
*E. melanophloia*	*Aspergillus niger*, *Rhizopus solani*	[75]
*E. microtheca*	*Aspergillus niger*, *Rhizopus solani*	[75]
*E. odorata*	*Candida albicans*, *Microsporum canis*, *Scopulariopsis brevicaulis*, *Trichophyton rubrum*, *Trichophyton soudanense*	[152]
*E. oleosa*	*Aspergillus ochraceus*, *Candida albicans*, *Mucor ramamnianus*, *Saccharomyces cerevisiae*	[155]
*E. robusta*	*Candida albicans*	[132]
*E. saligna*	*Candida albicans*	[73,132]
*E. olida*	*Candida albicans*	[17]
*E. platyphylla*	*Deightoniella torulosa*	[159]
*E. salmonophloia*	*Aspergillus ochraceus*, *Candida albicans*, *Mucor ramamnianus*, *Saccharomyces cerevisiae*	[155]
*E. salubris*	*Aspergillus ochraceus*, *Candida albicans*, *Mucor ramamnianus*, *Saccharomyces cerevisiae*	[155]
*E. sargentii*	*Aspergillus niger*, *Candida albicans*	[157]
*E. sideroxylon*	*Microsporum canis*	[152]
*E. smithii*	*Microsporum canis*, *Microsporum gypseum*, *Trichophyton mentagnophytes*, *Trichophyton rubrum*	[160]
*E. staigeriana*	*Candida albicans*	[17]
*E. tereticornis*	*Hansenula* spp., *Saccharomyces* spp., *Sporobolomyces*, *Torulopsis candida*	[134]
*E. urophylla*	*Aspergillus clavatus*, *Aspergillus niger*, *Chaetomium globosum*, *Cladosporium cladosporioides*, *Lenzites sulphureus*, *Myrothecium verrucaria*, *Penicillium citrinum*, *Phanerochaete chrysosporium*, *Phaeolus schweintizii*, *Trametes versicolor*, *Trichoderma viride*	[77]

**Table 4:** *Eucalyptus* spp. essential oils with acaricidal activities.

*Eucalyptus* spp.	Target Species	Reference
*E. approximans*	*Tetranychus urticae*	[166]
*E. bicostata*	*Tetranychus urticae*	[166]
*E. camaldulensis*	*Varroa destructor*	[71]
*E. citriodora*	*Boophilus microplus*, *Dermanyssus gallinae*, *Neoseiulus californicus*, *Tetranychus urticae*	[91,100,101,102]
*E. globulus*	*Boophilus microplus*	[91]
*E. maidenii*	*Tetranychus urticae*	[166]
*E. sideroxylon*	*Tetranychus urticae*	[166]
*E. staigeriana*	*Boophilus microplus*, *Dermanyssus gallinae*	[91,102]
*E. tereticornis*	*Amblyoma variegatum*	[134]

**Table 5:** *Eucalyptus* spp. essential oils with insecticidal activities.

*Eucalyptus* spp.	Target Species	Reference
*E. astringens*	*Callosobruchus maculatus*, *Ephestia cautela*, *Ephestia kuehniella*, *Rhyzopertha dominica*, *Tribolium castaneum*	[79,194]
*E. badjensis*	*Aedes aegypti*, *Haematobia irritans*	[195,196]
*E. badjensis x E. nitens*	*Aedes aegypti*, *Haematobia irritans*	[195,196]
*E. benthamii*	*Sitophilus zeamais*	[116]
*E. botryoides*	*Aedes aegypti*, *Haematobia irritans*	[195,196]
*E. camaldulensis*	*Aedes aegypti*, *Aedes albopictus*, *Atta sexdens rubropilosa*, *Ectomyelois ceratoniae*, *Ephestia cautela*, *Ephestia kuehniella*, *Pediculus humanus capitis*, *Sitophilus zeamais*, *Thyrinteina arnobia*	[63,64,65,66,67,72,78,79,80]
*E. cinerea*	*Aedes aegypti*, *Musca domestica*, *Pediculus humanus capitis*	[63,64,81,82,83]
*E. citriodora*	*Aedes aegypti*, *Anopheles gambia*, *Atta sexdens rubropilosa*, *Callosobruchus maculatus*, *Lutzomyia longipalpis*, *Nasutitermes corniger*, *Pediculus humanus capitis*, *Sitophilus zeamais*, *Thyrinteina arnobia*, *Tribolium castaneum*	[66,67,72,82,89,90,92,95,96,103,104,105]
*E. cloeziana*	*Atta sexdens rubropilosa*, *Thyrinteina arnobia*	[66,67]
*E. darlympleana*	*Aedes aegypti*, *Haematobia irritans*	[195,196]
*E. dorrigoensis*	*Aedes aegypti*, *Haematobia irritans*	[195,196]
*E. dundasii*	*Oryzaephilus surinamemsis*, *Rhyzopertha dominica*	[197]
*E. dunnii*	*Aedes aegypti*, *Blattella germanica*, *Pediculus humanus capitis*, *Sitophilus zeamais*	[63,64,110,116,129]
*E. elata*	*Haematobia irritans*	[195]
*E. fastigata*	*Aedes aegypti*, *Haematobia irritans*	[195,196]
*E. fraxinoides*	*Haematobia irritans*	[195]
*E. floribundi*	*Oryzaephilus surinamemsis*, *Rhyzopertha dominica*	[198]
*E. globulus*	*Aedes aegypti*, *Lutzomyia longipalpis*, *Musca domestica*, *Odontotermes assamensis*, *Pediculus humanus capitis*, *Sitophilus oryzae*, *Sitophilus zeamais*, *Tribolium castaneum*, *Tribolium confusum*	[63,64,72,95,110,111,112,113,116,117,120,121,122]
*E. grandis*	*Aedes aegypti*, *Atta sexdens rubropilosa*, *Blattella germanica*, *Pediculus humanus capitis*, *Thyrinteina arnobia*	[65,66,67,129,130]
*E. grandis x E. camaldulensis*	*Aedes aegypti*, *Blattella germanica*, *Pediculus humanus capitis*	[63,64,65,129]
*E. grandis x E. tereticornis*	*Aedes aegypti*, *Blattella germanica*, *Pediculus humanus capitis*	[63,64,65,129]
*E. gunnii*	*Aedes aegypti*, *Pediculus humanus capitis*	[63,64,110]
*E. lehmannii*	*Callosobruchus maculatus*, *Ephestia cautela*, *Ephestia kuehniella*, *Rhyzopertha dominica*, *Tribolium castaneum*	[79,194]
*E. leucoxylon*	*Ectomyelois ceratoniae*, *Ephestia cautela*, *Ephestia kuehniella*	[79,80]
*E. maculata*	*Atta sexdens rubropilosa*, *Thyrinteina arnobia*	[66,67]
*E. nobilis*	*Aedes aegypti*, *Haematobia irritans*	[195,196]
*E. oblicua*	*Haematobia irritans*	[195]
*E. polybractea*	*Aedes aegypti*, *Haematobia irritans*	[195,196]
*E. radiata*	*Aedes aegypti*, *Haematobia irritans*	[195,196]
*E. resinífera*	*Aedes aegypti*, *Haematobia irritans*	[195,196]
*E. robertsonii*	*Aedes aegypti*, *Haematobia irritans*	[195,196]
*E. rubida*	*Aedes aegypti*, *Haematobia irritans*	[195,196]
*E. rudis*	*Ectomyelois ceratoniae*, *Ephestia cautela*, *Ephestia kuehniella*	[79]
*E. saligna*	*Acanthoscelides obtectus*, *Aedes aegypti*, *Atta sexdens rubropilosa*, *Pediculus humanus capitis*, *Sitophilus zeamais*, *Sitotroga cerealella*, *Tribolium castaneum*, *Thyrinteina arnobia*	[63,64,66,67,82,116,131,133]
*E. sideroxylon*	*Aedes aegypti*, *Blattella germanica*, *Pediculus humanus capitis*	[63,64,110,129]
*E. smithii*	*Aedes aegypti*, *Haematobia irritans*	[195,196]
*E. staigeriana*	*Callosobruchus maculatus*, *Lutzomyia longipalpis*	[92,95]
*E. tereticornis*	*Aedes aegypti*, *Anopheles gambia*, *Pediculus humanus capitis*	[63,64,65,82,89]
*E. urophylla*	*Atta sexdens rubropilosa*, *Thyrinteina arnobia*	[66,67]
*E. viminalis*	*Aedes aegypti*, *Blattella germanica*, *Pediculus humanus capitis*, *Sitophilus zeamais*	[63,64,82,116,129]

**Table 6:** *Eucalyptus* spp. essential oils with herbicidal activities.

*Eucalyptus* spp.	Target Species	Reference
*E. brockwayii*	*Solanum elaeagnifolium*	[212,213]
*E. camaldulensis*	*Amaranthus hybridus*, *Portulaca oleracea*	[76]
*E. citriodora*	*Amaranthus viridis*, *Cassia occidentalis*, *Cucumis sativus*, *Echinochloa crus-galli*, *Oryza sativa*, *Sorghum bicolor*, *Triticum aestivum*	[26,54,99]
*E. dundasii*	*Solanum elaeagnifolium*	[212,213]
*E. erythrocorys*	*Phalaris canariensis*, *Sinapis arvensis*	[158]
*E. melliodora*	*Solanum elaeagnifolium*	[213]
*E. salubris*	*Solanum elaeagnifolium*	[212,213]
*E. spathulata*	*Solanum elaeagnifolium*	[212,213]
*E. urophylla*	*Lactuca sativa*	[214]

**Table 7:** *Eucalyptus* species typically used to produce medicinal essential oils.

*Eucalyptus* spp.	1,8-Cineole (%)	Reference
*E. camaldulensis*	80–90	[217,218]
*E. cineorifolia*	40–90	[217,218]
*E. dumosa*	33–70	[217,218]
*E. elaeophora*	60–80	[217,218]
*E. globulus*	60–85	[217,218]
*E. leucoxylon*	65–75	[217,218]
*E. oleosa*	45–52	[217,218]
*E. polybractea*	60–93	[217,218]
*E. radiata subsp. radiata var. cineole*	65–75	[217,218]
*E. sideroxylon*	60–75	[217,218]
*E. smithii*	70–80	[217,218]

## Supplementary Materials

Supplementary materials can be accessed at: http://www.mdpi.com/1420-3049/21/12/1671/s1. Table S1. Major chemical components for *Eucalyptus* spp. essential oils.
