# Supplementary material for: Chemical Variability and Biological Activities of Eucalyptus spp. Essential Oils
Source: Molecules. 2016 Dec 7;21(12):1671. doi: 10.3390/molecules21121671 (PMC6273930; doi:10.3390/molecules21121671)
Supplement: Supplementary file 1 [file molecules-21-01671-s001.pdf]

Supplementary Materials: Chemical Variability and Biological Activities of *Eucalyptus* spp. Essential Oils

Luiz Claudio Almeida Barbosa, Claudinei Andrade Filomeno and Robson Ricardo Teixeira

Table S1. Some common chemical components of essential oils extracted from leaves of *Eucalyptus* spp. [a].

Eucalyptus spp.	Origin	Components of *Eucalyptus* EOs	EOs Yields (%)	Reference
E. alba	Democratic Republic of Congo	β-pinene (25.3%), β-terpineol (13.6%)	0.22 [b]	[1]
	Nigeria	α-thujene (32.9%), 1,8-cineole (13.3%), *p*-cymene (12.9%)	0.28	[2]
E. approximans	Australia	1,8-cineole (61.1%), limonene (14.5%), α-pinene (9.7%)	-	[3]
E. astringens	Tunisia	1,8-cineole (60.0%), *trans*-pinocarveol (8.9%), α-pinene (6.7%)	1.20	[4]
	Tunisia	1,8-cineole (43.7%), α-pinene (21.3%)	3.80	[5,6]
	Tunisia	α-pinene (29.8%), 1,8-cineole (17.3%), viridiflorol (11.2%)	1.49	[7,8]
E. badjensis	Argentina	1,8-cineole (71.7%), β-eudesmol (7.8%)	-	[9,10]
E. badjensis x E. nitens	Argentina	1,8-cineole (82.8%), limonene (5.9%)	-	[9,10]
E. benthamii	Brazil	α-pinene (54.0%), viridiflorol (17.1%), 1,8-cineole (9.9%)	2.60	[11]
E. bicostata	Australia	1,8-cineole (63.0%), α-pinene (14.4%), limonene (10.9%)	-	[3]
	Tunisia	1,8-cineole (81.3%), *trans*-pinocarveol (4.5%), pinocarvone (3.9%)	2.00	[4]
	Tunisia	1,8-cineole (68.0%), globulol (5.4%)	3.00	[6,12]
E. botryoides	Argentina	*p*-cymene (19.9%), α-eudesmol (15.0%), 1,8-cineole (13.3%)	-	[9,10]
	Morocco	1,8-cineole (18.4%), *p*-cymene (12.6%), α-pinene (9.4%)	0.40	[13,14]
E. brockwayii	Australia	1,8-cineole (37.1%), *trans*-pinocarveol (7.0%), globulol (6.9%)	-	[15]
	Australia	α-pinene (31.1%), isopentyl isovalerate (20.2%), 1,8-cineole (16.9%)	-	[16]
E. camaldulensis	Argentina	1,8-cineole (19.1%), *p*-cymene (17.9%), β-phellandrene (16.3%)	0.38	[17-19]
	Brazil	1,8-cineole (52.8%), limonene (14.2%), γ-terpinene (6.8%), α-pinene (6.1%)	0.63	[20,21]
	Brazil	1,8-cineole (44.8%), α-phellandrene (22.9%), *p*-cymene (9.8%)	3.00	[22]
	Democratic Republic of Congo	1,8-cineole (58.9%), myrtenol (4.3%), myrtenal (3.5%)	0.30 [b]	[1]
	Egypt	1,8-cineole (60.3%), α-pinene (13.6%), γ-terpinene (8.8%)	-	[23]
	India	1,8-cineole (8.7%), α-phellandrene (27.5%), β-pinene (23.5%), *m*-cymene (9.5%)	1.97 [b]	[24]
	Iran	1,8-cineole (74.7%)	-	[25]
	Kenya	1,8-cineole (18.9%), α-cadinol (6.4%), β-phellandrene (2.6%)	-	[26]
	Nigeria	1,8-cineole (70.4%), β-pinene (9.0%), α-pinene (8.8%)	0.26	[2]
	Northern Cyprus	1,8-cineole (19.0%), β-caryophyllene (11.6%), carvacrol (9.1%)	-	[27]
	Pakistan	linalool (17.0%), 1,8-cineole (16.1%), *p*-cymene (12.2%)	1.90	[28]
	Spain	spathulenol (41.5%), *p*-cymene (21.9%)	0.71	[29]
	Taiwan	1,8-cineole (29.6%), limonene (15.2%), β-pinene (9.9%), α-pinene (9.7%)	3.48	[30]
	Taiwan	α-pinene (22.5%), *p*-cymene (21.7%), α-phellandrene (20.1%), 1,8-cineole (9.5%)	0.57	[31]
	Tunisia	1,8-cineole (20.6%), α-pinene (16.5%)	0.76–1.42	[8,32]
E. cinerea	Argentina	1,8-cineole (88.5%), α-terpineol (9.0%), α-pinene (2.0%)	-	[33]
	Argentina	1,8-cineole (79.8%), α-terpinyl acetate (8.2%)	2.48	[17,18]
	Argentina	1,8-cineole (62.1%), *p*-cymene (11.2%)	-	[34]
	Argentina	1,8-cineole (56.9%), α-pinene (6.4%)	-	[35]
	Brazil	1,8-cineole (83.6%), α-terpinyl acetate (5.4%), α-pinene (5.0%)	3.56–5.02	[36]
	Brazil	1,8-cineole (75.7%), α-terpineol (9.7%), α-pinene (6.2%)	6.07	[37]

Table S1. *Cont*.

	Tunisia	1,8-cineole (79.2%), α-terpinyl acetate (5.4%), α-pinene (4.1%)	3.00	[4]
	Tunisia	1,8-cineole (70.4%), α-terpineol (10.3%)	3.90	[12]
E. citriodora	Argentina	citronellal (76.0%), iso-isopulegol (9.0%), citronellyl acetate (7.3%)	-	[34]
	Australia	citronellal (68.9%), citronellol (7.6%), isopulegol (7.4%)	-	[38]
	Benin	citronellal (52.8%), citronellol (20.0%), citronellyl acetate (9.0%)	4.60	[39,40]
	Brazil	citronellal (94.9%), citronellyl acetate (2.6%), *trans* caryophyllene (2.5%)	-	[41]
	Brazil	citronellal (89.6%), citronellyl acetate (3.3%), 1,8-cineole (2.9%)	-	[42]
	Brazil	citronellal (82.3%), citronellyl acetate (7.8%), neothujan-3-ol (6.8%)	4.00	[43]
	Brazil	citronellal (76.0%), *neo*-*iso*-3-thujanol (11.8%)	0.66	[20,21]
	Brazil	citronellal (71.8%), isopulegol (4.3%)	-	[44,45]
	Brazil	citronellal (71.1%), citronellol (8.8%)	-	[46]
	Brazil	citronellal (67.5%), citronellol (6.9%), menthol (6.1%)	-	[47]
	Brazil	citronellal (64.9%), *iso*-isopulegol (10.2%), citronellol (8.3%)	2.10	[48]
	Brazil	citronellal (61.8%), isopulegol (15.5%), β-citronellol (7.9%)	-	[49]
	China	citronellal (65.9%), citronellol (10.5%), 1,8-cineole (3.0%)	-	[50,51]
	China	citronellal (55.3%), citronellol (8.3%)	-	[52]
	Colombia	citronellal (49.3%), citronellol (13.0%), isopulegol (12.9%)	0.70	[53]
	Colombia	citronellal (40.0%), isopulegol (14.6%), citronellol (13.0%)	-	[54,55]
	Democratic Republic of Congo	citronellal (72.7%), citronellol (6.3%), eugenol (3.5%)	1.63 [b]	[1]
	India	citronellal (52.2%), citronellol (12.3%), isopulegol (11.9%)	0.60	[56]
	India	citronellal (48.3%), citronellol (21.9%), *iso*-isopulegol (12.7%)	2.36–4.80	[57]
	Indonesia	citronellal (90.1%), citronellol (4.3%)	-	[58]
	Kenya	1,8-cineole (11.2%), β-pinene (3.2%), terpinen-4-ol (3.1%)	-	[26]
	Pakistan	citronellal (22.3%), citronellol (20.0%)	1.82	[28]
	South Korea	citronellal (73.0%), isopulegol (6.7%)	-	[59]
	Taiwan	citronellal (49.5%), citronellol (11.9%), *iso*-isopulegol (10.4%)	1.89	[30]
	Tunisia	1,8-cineole (54.1%), α-pinene (23.6%)	3.30	[13,14]
E. cloeziana	Brazil	myrcene (31.8%), β-pinene (29.5%), 9-epicaryophyllene (6.6%)	0.75	[43]
	Brazil	α-pinene (76.1%), α-terpineol (3.8%), β-caryophyllene (2.3%)	0.17	[20,21]
	Brazil	α-pinene (27.5%), β-eudesmol (11.3%), α-eudesmol (10.8%), spathulenol (10.4%)	0.13	[22]
E. crebra	Pakistan	α-pinene (16.0%), β-phellandrene (14.3%)	1.84	[28]
E. darlympleana	Argentina	1,8-cineole (80.3%), *p*-cymene (5.6%)	-	[9,10]
E. deglupta	Democratic Republic of Congo	1,8-cineole (35.7%), cryptone (25.4%), myrtenol (7.4%), α-phellandrene (7.2%)	0.15 [b]	[1]
	Nigeria	*E*-β-nerolidol (34.8%), α-pinene (24.7%)	0.20	[2]
E. diversifolia	Tunisia	1,8-cineole (37.0%), *trans*-pinocarveol (7.0%)	0.80	[5,60]
E. dives	Australia	piperitone (40.5%), α-phellandrene (17.4%), *p*-cymene (8.5%)	2.97	[61]
	South Africa	piperitone (73.5%), terpinen-4-ol (7.9%)	-	[62]
E. dorrigoensis	Argentina	1,8-cineole (74.7%), viridiflorol (7.4%)	-	[9,10]

Table S1. *Cont*.

E. dundasii	Australia	1,8-cineole (80.1%), *trans*-pinocarveol (4.3%)	-	[15]
	Australia	1,8-cineole (65.5%), α-pinene (19.9%)	-	[16]
	Iran	1,8-cineole (54.2%), *p*-cymene (12.4%), α-thujene (11.4%)	1.53	[63]
E. dunnii	Argentina	1,8-cineole (49.6%), γ-terpinene (11.9%), *p*-cymene (7.0%)	-	[64,65]
	Argentina	1,8-cineole (48.5%), γ-terpinene (13.0%), α-pinene (5.5%)	0.62	[17,18]
	Brazil	1,8-cineole (53.5%), α-pinene (21.5%), viridiflorol (8.3%)	2.00	[11]
E. elata	Argentina	α-phellandrene (16.0%), β-phellandrene (14.5%), *p*-cymene (14.8%), *cis-p*-menth-2-en-1-ol (12.2%)	-	[9]
E. erythrocorys	Tunisia	1,8-cineole (54.8%), α-pinene (7.8%)	0.90	[66]
E. fastigata	Argentina	*p*-cymene (37.6%), 1,8-cineole (14.7%), β-phellandrene (9.2%)	-	[9,10]
E. fraxinoides	Argentina	*p*-cymene (35.5%), 1,8-cineole (13.4%), β-phellandrene (8.8%)	-	[9]
E. floribundi	Iran	1,8-cineole (58,0%), α-pinene (26.2%)	-	[67]
E. globulus	Algeria	1,8-cineole (55.3%), spathulenol (7.4%), α-terpineol (5.5%)	2,53	[68]
	Argentina	1,8-cineole (77.9%), α-terpineol (6.0%)	2.25	[17,18]
	Argentina	1,8-cineole (76.7%), α-pinene (11.1%)	1.66	[17,65]
	Argentina	1,8-cineole (52,3-62.1%)	1.31–1.49	[69]
	Australia	1,8-cineole (90.0%), α-pinene (2.2%)	-	[70]
	Australia	1,8-cineole (81.1%), limonene (7.6%), α-pinene (4.0%)	-	[71]
	Brazil	1,8-cineole (90.0%), tricyclene (3.0%)	-	[72]
	Brazil	1,8-cineole (85.8%), α-pinene (9.9%)	-	[41]
	Brazil	1,8-cineole (83.9%), limonene (8.2%), α-pinene (4.2%)	-	[45,73]
	Brazil	1,8-cineole (77.5%), α-pinene (14.2%)	3.10	[11]
	Democratic Republic of Congo	1,8-cineole (44.3%), camphene (23.1%), α-pinene (9.3%), globulol (7.3%)	1.87 [b]	[1]
	Egypt	1,8-cineole (21.4%), *o*-cimene (21.4%), α-pinene (6.7%), spathulenol (6.3%)	-	[74]
	Ethiopia	1,8-cineole (63.0%), α-pinene (16.1%)	-	[75]
	India	1,8-cineole (81.9%), limonene (6.6%)	-	[76]
	India	1,8-cineole (68.8%), α-pinene (2.8%)	-	[77]
	India	1,8-cineole (66.3%), *cis*-ocymene (21.3%), α-terpinyl acetate (3.4%)	-	[78]
	India	1,8-cineole (44.4%), limonene (17.8%), *p*-cymene (9.5%)	-	[79]
	India	1,8-cineole (33.6%), α-pinene (14.2%), limonene (10.1%)	-	[80]
	Indonesia	1,8-cineole (86.5%), α-pinene (4.7%)	-	[58]
	Iran	1,8-cineole (84.5%), limonene (8.50%)	-	[81]
	Iran	1,8-cineole (47.2%), spathulenol (18.1%), α-pinene (9.6%)	-	[82]
	Italy	1,8-cineole (84.9%), α-pinene (5.6%), *p*-cymene (5.3%)	-	[83]
	Kenya	1,8-cineole (17.2%), α-pinene (7.1%), spathulenol (6.5%)	-	[26]
	Montenegro	1,8-cineole (85.8%), α-pinene (7.2%), β-myrcene (1.5%)	1.80 [b]	[84]
	Morocco	1,8-cineole (22.4%), limonene (7.0%), solanone (6.1%), β-pinene (5.2%)	1.21	[85]

Table S1. *Cont*.

	Pakistan	1,8-cineole (56.5%), limonene (28.0%)	1.89	[28]
	Spain	1,8-cineole (63.8%), α-pinene (16.1%)	-	[86]
E. gracilis	Tunisia	1,8-cineole (71.6%), α-pinene (18.2%)	7.30	[87]
E. grandis	Argentina	α-pinene (52.7%), 1,8-cineole (18.4%), *p*-cymene (8.7%)	0.36	[19,64,88]
	Brazil	*p*-cymene (59.6%), γ-terpinene (29.2%)	0.26	[22]
	Brazil	α-pinene (40.6%), γ-terpinene (16.3%), *p*-cymene (13.1%)	0.31	[20,21]
	Brazil	γ-terpinene (16.8%), *o*-cymene (16.7%), β-pinene (11.5%)	2.00	[45]
	Taiwan	1,8-cineole (19.8%), α-terpinyl acetate (12.8%), α-pinene (11.4%)	3.01	[30]
E. grandis x E. camaldulensis	Argentina	1,8-cineole (49.7%), α-pinene (30.7%)	0.54	[17–19,64]
E. grandis x E. tereticornis	Argentina	1,8-cineole (63.0%), α-pinene (22.8%)	0.88	[17–19,64]
E. grandis x E. urophylla	Brazil	α-pinene (53.4%), 1,8-cineole (33.0%)	1.56	[22]
	Brazil	α-pinene (36.8%), 1,8-cineole (33.7%)	-	[89]
E. gunnii	Argentina	1,8-cineole (26.7%), *p*-cymene (13.6%)	-	[65]
	Argentina	1,8-cineole (18.0%), *p*-cymene (12.3%), spathulenol (12.3%)	0.21	[17,18]
E. lehmannii	Tunisia	1,8-cineole (56,6%), α-pinene (17,6%)	3.60	[5]
	Tunisia	1,8-cineole (49,1%), α-pinene (26,4%), α-terpinyl acetate (5,6%)	2.80	[4]
	Tunisia	1,8-cineole (34.6%), α-pinene (31.6%)	1.74–2.52	[7,8]
E. leucoxylon	Tunisia	1,8-cineole (77.8%), α-pinene (5.9%), *trans*-pinocarveol (3.2%)	1.60	[4]
	Tunisia	α-pinene (32.7%), 1,8-cineole (17.6%), globulol (14.7%)	0.58–0.93	[8,32]
E. maculata	Brazil	α-pinene (39.4%), β-caryophyllene (10.3%)	0.07	[20,21]
	Brazil	α-pinene (68.1%), guaiol (8.8)	-	[89]
E. maidenii	Australia	1,8-cineole (59.8%), α-pinene (17.2%), limonene (5.5%)	-	[3]
	Tunisia	1,8-cineole (83.6%), globulol (3.6%), *trans*-pinocarveol (3.4%)	1.50	[4]
	Tunisia	1,8-cineole (57.8%), α-pinene (7.3%)	3.30	[6,12]
E. melanophloia	Pakistan	α-pinene (16.0%), β-phellandrene (14.3%)	1.73	[28]
E. melliodora	Australia	1,8-cineole (54.7%), α-terpineol (9.6%), globulol (3.4%)	-	[15]
E. microcorys	Brazil	1,8-cineole (86.7%), α-terpineol (3.9%)	2.50	[43]
	Brazil	1,8-cineole (66.2%), α-pinene (9.9%)	1.70	[22]
E. microtheca	Pakistan	α-pinene (31.4%), citrinyl acetate (13.2%), *p*-cymene (12.4%)	1.84	[28]
E. nobilis	Argentina	1,8-cineole (30.4%), *p*-cymene (18.2%), α-pinene (12.9%), viridiflorol (11.3%)	-	[9,10]
E. oblicua	Argentina	*p*-cymene (25.4%), piperitone (23.2%)	-	[9]
E. odorata	Tunisia	cryptone (20.9%), *p*-cymene (16.7%)	1.70	[6,12]
E. oleosa	Tunisia	1,8-cineole (41.2%), α-pinene (21.8%)	4.90	[87]
E. olida	Australia	*(E)*-methyl cinnamate (99.4%)	3.12	[61]
E. ovata	Morocco	1,8-cineole (41.6%), *trans*-pinocarveol (13.8%), α-pinene (13.5%)	1.20	[13,14]
E. paniculata	Brazil	spathulenol (22.6%), *p*-cymene (19.4%), globulol (10.4%)	0.60	[22]
E. pellita	Cuba	α-pinene (27.2%), limonene (23.8%), 1,8-cineole (19.0%)	0.89	[90]

Table S1. *Cont*.

E. pilularis	Brazil	*p*-cymene (38.0%), cyclocolorenone (14.0%)	0.73	[22]
E. phoenicea	Brazil	*α*-pinene (44.6%), *β*-pinene (31.2%)	-	[89]
E. platyphylla	Ivory Coast	limonene (26.4%), 1,8-cineole (20.0%), *γ*-terpinene (18.9%)	-	[91]
E. platypus	Tunisia	1,8-cineole (22.5%), spathulenol (11.2%), *α*-pinene (9.4%)	1.90	[5,6]
E. polybractea	Argentina	1,8-cineole (85.0%), *p*-cymene (4.1%)	-	[9,10]
E. propinqua	Democratic Republic of Congo	1,8-cineole (32.4%), *α*-pinene (20.3%), *β*-pinene (9.3%), *α*-terpineol (7.4%)	0.65 [b]	[1]
E.punctata	Brazil	1,8-cineole (55.6%), *α*-pinene (27.3%)	-	[89]
E. radiata	Argentina	1,8-cineole (68.4%), *α*-terpineol (12.4%), limonene (7.3%)	-	[9,10]
	Australia	limonene (68.5%), *α*-terpineol (8.6%), *α*-terpenyl acetate (6.1%)	-	[86]
	Indonesia	1,8-cineole (82.7%), *α*-terpineol (7.0%), *α*-pinene (3.7%)	-	[58]
E. resinifera	Argentina	1,8-cineole (58.6%), *p*-cymene (12.0%), *α*-pinene (10.0%)	-	[9,10]
E. robertsonii	Argentina	1,8-cineole (62.0%), *α*-terpineol (8.6%)	-	[9,10]
E. robusta	Brazil	*α*-phellandrene (36.6%), *α*-pinene (16.6%), *p*-cymene (14.8%), β-pinene (11.8%)	0.34	[22]
	Brazil	*α*-pinene (73.0%), limonene (8.3%)	0.20	[92]
	Democratic Republic of Congo	*p*-cymene (27.3%), myrtenal (12.8%), β-pinene (6.3%), *α*-terpineol (6.3%)	0.13 [b]	[1]
E. rubida	Argentina	1,8-cineole (82.5%), limonene (4.1%)	-	[9,10]
E. rudis	Tunisia	1,8-cineole (19.9%), *α*-pinene (14.5%)	0.74–2.09	[8]
E. saligna	Argentina	1,8-cineole (93.2%)	-	[93]
	Argentina	1,8-cineole (93.2%), limonene (3.3%)	-	[34]
	Argentina	1,8-cineole (34.0%), *p*-cymene (21.3%), *γ*-terpinene (20.10%), *α*-pinene (13.0%)	0.36	[17,18]
	Brazil	1,8-cineole (45.2%), *p*-cymene (34.4%), *α*-pinene (12.8%)	0.50	[11]
	Brazil	*p*-cymene (25.6%), *α*-terpineol (9.3%), *α*-camphonelal (8.0%), 1,8-cineole (6.2%)	0.50	[43]
	Brazil	*α*-pinene (92.3%)	1.42	[22]
	Brazil	*α*-pinene (45.1%), *p*-cymene (22.5%), *α*-pinene oxide (11.3%)	0.40	[92]
	Brazil	*α*-pinene (34.8%), geranyl formate (10.3%)	-	[89]
	Brazil	*α*-pinene (25.9%), *p*-cymene (24.4%), *γ*-terpinene (24.6%)	0.19	[20,21]
	Democratic Republic of Congo	1,8-cineole (61.3%), limonene (10.1%), *p*-cymene (7.2%)	0.78 [b]	[1]
	Kenya	*α*-pinene (24.4%), 1,8-cineole (24.3%), *o*-cimene (9.9%), *α*-terpineol (8.8%)	0.38	[94]
	Nigeria	*α*-thujene (63.8%), 1,8-cineole (12.3%)	0.30	[2]
E. salmonophloia	Tunisia	1,8-cineole (59.3%), *α*-pinene (10.7%)	4.60	[87]
E. salubris	Tunisia	1,8-cineole (71.3%), *trans*-pinocarveol (6.0%)	4.80	[87]
E. sargentii	Iran	1,8-cineole (55.5%), *α*-pinene (21.0%), aromadendrene (6.5%)	1.40	[95]
E. sideroxylon	Argentina	1,8-cineole (91.3%), *α*-terpineol (2.6%)	1.65	[17,18,64,65]
	Australia	1,8-cineole (54.4%), limonene (11.9%), *α*-pinene (8.2%)	-	[3]
	Tunisia	1,8-cineole (80.8%), *α*-pinene (5.8%), limonene (3.3%)	3.00	[4]
	Tunisia	1,8-cineole (69.2%), *α*-pinene (6.9%)	2.70	[6,12]
E. smithii	Argentina	1,8-cineole (78.5%), limonene (5.9%)	-	[9,10]

Table S1. *Cont*.

	Brazil	1,8-cineole (72.2%), α-terpineol (7.5%)	-	[96]
E. staigeriana	Australia	1,8-cineole (34.8%), neral (10.8%), geranial (10.8%)	2.13	[61]
	Brazil	geranial (16.0%), geraniol (14.8%)	-	[97]
	Brazil	limonene (72.9%), 1,8-cineole (9.5%), *o*-cymene (4.6%)	-	[98]
	Brazil	limonene (28.8%), geranial (15.2%), neral (12.2%)	-	[42]
	Brazil	limonene (28.8%), *E*-citral (14.2%), *Z*-citral (10.8%)	-	[45,99]
	Brazil	limonene (24.8%), *E*-citral (15.0%), *Z*-citral (11.4%), α-terpinolene (10.8%)	-	[41]
	China	β-thujene (25.5%), geraniol (21.7%), β-citral (17.8%)	-	[52]
E. tereticornis	Argentina	1,8-cineole (37.5%), *p*-cymene (22.0%), γ-terpinene (10.8%)	-	[34]
	Argentina	β-phellandrene (22.6%), 1,8-cineole (18.6%), *p*-cymene (14.5%), α-phellandrene (9.4%)	0.60	[17-19]
	Benin	*p*-cymene (31.1%), β-phellandrene (9.7%)	-	[100]
	Benin	*p*-cymene (16.7%), caryophyllene oxide (14.2%), spathulenol (13.5%), cryptone (11.4%)	1.00	[39]
	Brazil	β-pinene (22.4%), 1,8-cineole (19.3%), α-pinene (13.6%), α-phellandrene (10.3%)	2.30	[22]
		β-pinene (20.4%), 1,8-cineole (17.3%), *p*-cymene (14.8%), α-pinene (11.4%)	-	[89]
	Democratic Republic of Congo	*p*-cymene (28.6%), cryptone (17.8%), α-pinene (8.3%)	0.45 b	[1]
E. torelliana	Brazil	α-pinene (69.6%), *E*-caryophyllene (7.4%)	-	[89]
E. urophylla	Brazil	1,8-cineole (65.4%), α-terpinyl acetate (8.4%)	0.77	[22]
	Brazil	1,8-cineole (53.1%), α-pinene (8.0%)	0.29	[20,21]
	Democratic Republic of Congo	1,8-cineole (57.7%), α-pinene (10.1%), globulol (4.4%)	0.53 b	[1]
	Taiwan	γ-terpinene (26.2%), *p*-cymene (22.3%), 1,8-cineole (13.9%)	3.14	[30]
E. viminalis	Argentina	1,8-cineole (85.0%), globulol (2.5%)	-	[64]
	Argentina	1,8-cineole (85.0%), aromadendrene (2.0%)	1.46	[17,18]
	Argentina	1,8-cineole (46.9%), γ-terpinene (23.2%), *p*-cymene (17.4%)	-	[34]
	Brazil	1,8-cineole (77.1%), α-pinene (14.8%)	2.40	[11]

a The compounds are listed according to their decreasing quantities; b Fresh leaves; (-): not reported.

References

1. Cimanga, K.; Kambu, K.; Tona, L.; Apers, S.; de Bruyne, T.; Hermans, N.; Totté, J.; Pieters, L.; Vlietinck, A.J. Correlation between chemical composition and antibacterial activity of EOs of some aromatic medicinal plants growing in the Democratic Republic of Congo. *J. Ethnopharmacol.* **2002**, *79*, 213–220.
2. Oyedeji, A.O.; Ekundayo, O.; Olawore, O.N.; Adeniyi, B.A.; Koenig, W.A. Antimicrobial activity of the EOs of five *Eucalyptus* species growing in Nigeria. *Fitoterapia* **1999**, *70*, 526–528.
3. Roh, H.S.; Lee, B.H.; Park, C.G. Acaricidal and repellent effects of myrtacean EOs and their major constituents against *Tetranychus urticae* (Tetranychidae). *J. Asia-Pac. Entomol.* **2013**, *16*, 245–249.
4. Sebei, K.; Sakouhi, F.; Herchi, W.; Khouja, M.L.; Boukhchina, S. Chemical composition and antibacterial activities of seven *Eucalyptus* species EOs leaves. *Biol. Res.* **2015**, *48*, 7.
5. Elaissi, A.; Medini, H.; Khouja, M.L.; Simmonds, M.; Lynene, F.; Farhat, F.; Chemli, R.; Harzallah-Skhiri, F. Variation in volatile leaf oils of eleven *Eucalyptus* species Harvested from Korbous Arboreta (Tunisia). *Chem. Biodivers.* **2010**, *7*, 1841–1854.
6. Elaissi, A.; Rouis, Z.; Salem, N.A.B.; Mabrouk, S.; Salem, Y.B.; Salah, K.B.H.; Aouni, M.; Farhat, F.; Chemli, R.; Harzallah-Skhiri, F.; Khouja, M.L. Chemical composition of 8 *Eucalyptus* species' EOs and the evaluation of their antibacterial, antifungal and antiviral activities. *Complement. Altern. Med.* **2012**, *12*, 81.
7. Hamdi, S.H.; Hedjal-Chebheb, M.; Kellouche, A.; Khouja, M.L.; Boudabous, A.; Jemaa, J.M.B. Management of three pests' population strains from Tunisia and Algeria using *Eucalyptus* essential oils. *Ind. Crops Prod.* **2015**, *74*, 551–556.
8. Jemaa, J.M.B.; Haouel, S.; Bouaziz, M.; Khouja, M.L. Seasonal variations in chemical composition and fumigant activity of five *Eucalyptus* EOs against three moth pests of stored dates in Tunisia. *J. Stored Prod. Res.* **2012**, *48*, 61–67.
9. Juan, L.; Lucia, A.; Zerba, E.; Harrand, L.; Marco, M.; Masuh, H. Chemical composition and fumigant toxicity of the EOs from 16 species of *Eucalyptus* against *Haematobia irritans* (L.) (Diptera: Muscidae) adults. *J. Econ. Entomol.* **2011**, *104*, 1087–1092.
10. Lucia, A.; Juan, L.W.; Zerba, E.N.; Harrand, L.; Marcó, M.; Masuh, H.M. Validation of models to estimate the fumigant and larvicidal activity of *Eucalyptus* EOs against *Aedes aegypti* (Diptera: Culicidae). *Parasitol. Res.* **2012**, *110*, 1675–1686.
11. Mossi, A.J.; Astolfi, V.; Kubiak, G.; Lerin, L.; Zanella, C.; Toniazzo, G.; Oliveira, D.; Treichel, H.; Devilla, I.A.; Cansiana, R.; Restello, R. Insecticidal and repellency activity of essential oil of *Eucalyptus* sp. against *Sitophilus zeamais* Motschulsky (Coleoptera, Curculionidae). *J. Sci. Food Agric.* **2011**, *91*, 273–277.
12. Elaissi, A.; Marzouki, H.; Medini, H.; Khouja, M.L.; Farhat, F.; Lynene, F.; Harzallah-Skhiri, F.; Chemli, R. Variation in volatile leaf oils of 13 *Eucalyptus* species harvested from Souinet Arboreta (Tunisia). *Chem. Biodivers.* **2010**, *7*, 909–921.
13. Elaissi, A.; Salah, K.H.; Mabrouk, S.; Larbi, K.M.; Chemli, R.; Harzallah-Skhiri, F. Antibacterial activity and chemical composition of 20 *Eucalyptus* species' EOs. *Food Chem.* **2011**, *129*, 1427–1434.
14. Elaissi, A.; Medini, H.; Simmonds, M.; Lynen, F.; Farhat, F.; Chemli, R.; Harzallah-Skhiri, F.; Khouja, M.L. Variation in volatile leaf oils of seven *Eucalyptus* species Harvested from Zerniza Arboreta (Tunisia). *Chem. Biodivers.* **2011**, *8*, 362–372.
15. Zhang, J.; An, M.; Wu, H.; Liu, D.L.; Stanton, R. Phytotoxic Activity and Chemical Composition of Aqueous Volatile Fractions from *Eucalyptus* Species. *PLoS ONE* **2014**, *9*, e93189.
16. Zhang, J.; An, M.; Wu, H. Chemical composition of EOs of four *Eucalyptus* species and their phytotoxicity on silver leaf nightshade (*Solanum elaeagnifolium* Cav.) in Australia. *Plant Growth Regul.* **2012**, *68*, 231–237.
17. Lucia, A.; Licastro, S.; Zerba, E.; Masuh, H. Yield, chemical composition, and bioactivity of EOs from 12 species of *Eucalyptus* on *Aedes aegypti* larvae. *Entomol. Exp. Appl.* **2008**, *129*, 107–114.
18. Lucia, A.; Licastro, S.; Zerba, E.; Gonzalez, A.P.; Masuh, H. Sensitivity of *Aedes aegypti* adults (Diptera: Culicidae) to the vapors of *Eucalyptus* EOs. *Bioresour. Technol.* **2009**, *100*, 6083–6087.
19. Toloza, A.; Lucia, A.; Zerba, E.; Masuh, H.; Picollo, M.I. Interspecific hybridization of *Eucalyptus* as a potential tool to improve the bioactivity of EOs against permethrin-resistant head lice from Argentina. *Bioresour. Technol.* **2008**, *99*, 7341–7347.

20. Batista-Pereira, L.G.; Fernandes, J.B.; Silva, M.F.G.F.; Vieira, P.C.; Bueno, O.C.; Correêa, A.G. Electrophysiological responses of *Atta sexdens rubropilosa* workers to EOs of *Eucalyptus* and its chemical composition. *Z. Naturforsch.* **2006**, *61*, 749–755.
21. Batista-Pereira, L.G.; Fernandes, J.B.; Correa, A.G.; da Silva, M.F.G.F.; Vieira, P.C. Electrophysiological Responses of *Eucalyptus* Brown Looper *Thyrinteina arnobia* to EOs of Seven *Eucalyptus* Species. *J. Braz. Chem. Soc.* **2006**, *17*, 555–561.
22. Filomeno, C.A.; Barbosa, L.C.A.; Pereira, J.L.; Pinheiro, A.L.; Fidencio, P.H.; Montanari, R.M. The chemical diversity of *Eucalyptus* spp. essential oils from plants grown in Brazil. *Chem. Biodivers.* **2016**, doi:10.1002/cbdv.201600097.
23. Salem, M.Z.M.; Zidan, Y.E.; Mansour, M.M.A.; El Hadidi, N.M.N.; Abo Elgat, W.A.A. Antifungal activities of two essential oils used in the treatment of three commercial woods deteriorated by five common mold fungi. *Int. Biodeterior. Biodegrad.* **2016**, *106*, 88–96.
24. Debbarma, J.; Kishore, P.; Nayak, B.B.; Kannuchamy, N.; Gudipati, V. Antibacterial activity of ginger, *Eucalyptus* and sweet orange peel EOs on fish-borne bacteria. *J. Food Process. Preserv.* **2013**, *37*, 1022–1030.
25. Ghasemi, V.; Moharramipour, S.; Tahmasbi, G. Biological activity of some plant EOs against *Varroa destructor* (Acari: Varroidae), an ectoparasitic mite of *Apis mellifera* (Hymenoptera: Apidae). *Exp. Appl. Acarol.* **2011**, *55*,147–154.
26. Karemu, C.K.; Ndung'u, M.W.; Githua, M. Repellent effects of EOs from selected *Eucalyptus* species and their major constituents against *Sitophilus zeamais* (Coleoptera: Curculionidae). *Int. J. Trop. Insect Sci.* **2013**, *33*, 188–194.
27. Akin, M.; Aktumsek, A.; Nostro, A. Antibacterial activity and composition of the EOs of *Eucalyptus camaldulensis* Dehn. and *Myrtus communis* L. growing in Northern Cyprus. *Afr. J. Biotechnol.* **2010**, *9*, 531–535.
28. Ghaffar, A.; Yameen, M.; Kiran, S.; Kamal, S.; Jalal, F.; Munir, B.; Saleem, S.; Rafiq, N.; Ahmad, A.; Saba, I.; Jabbar, A. Chemical Composition and *in-vitro* Evaluation of the Antimicrobial and Antioxidant Activities of Essential Oils Extracted from Seven *Eucalyptus* Species. *Molecules* **2015**, *20*, 20487–20498.
29. Verdeguer, M.; Blazquez, M.A.; Boira, H. Phytotoxic effects of Lantana camara, *Eucalyptus camaldulensis* and *Eriocephalus africanus* EOs in weeds of Mediterranean summer crops. *Biochem. Syst. Ecol.* **2009**, *37*, 362–369.
30. Su, Y.C.; Ho, C.L.; Wang, E.I.; Chang, S.T. Antifungal activities and chemical compositions of EOs from leaves of four *Eucalyptus*. *Taiwan J. Sci.* **2006**, *21*, 49–61.
31. Cheng, S.S.; Huang, C.G.; Chen, Y.J.; Yu, J.J.; Chen, W.J.; Chang, S.T. Chemical compositions and larvicidal activities of leaf EOs from two *Eucalyptus* species. *Bioresour. Technol.* **2009**, *100*, 452–456.
32. Jemaa, J.M.B.; Haouel, S.; Khouja, M.L. Efficacy of *Eucalyptus* EOs fumigant control against *Ectomyelois ceratoniae* (Lepidoptera: Pyralidae) under various space occupation conditions. *J. Stored Prod. Res.* **2013**, *53*, 67–71.
33. Rossi, Y.E.; Palacios, S.M. Insecticidal toxicity of *Eucalyptus cinerea* essential oil and 1,8-cineole against *Musca domestica* and possible uses according to the metabolic response of flies. *Ind. Crops Prod.* **2015**, *63*, 133–137.
34. Toloza, A.C.; Zygadlo, J.; Mougabure, C.G.; Biurrun, F.; Zerba, E. Picollo, M.I. Fumigant and repellent properties of EOs and component compounds against permethrin-resistant *Pediculus humanus capitis* (Anoplura: Pediculidae) from Argentina. *J. Med. Entomol.* **2006**, *43*, 889–895.
35. Palacios, S.M.; Bertoni, A.; Rossi, T.; Santander, R.; Urzúa, A. Efficacy of EOs from Edible Plants as Insecticides against the House Fly, *Musca Domestica* L. *Molecules* **2009**, *14*, 1938–1947.
36. Silva, S.M.; Abe, S.Y.; Murakami, F.S.; Frensch, G.; Marques, F.A.; Nakashima, T. EOs from Different Plant Parts of *Eucalyptus cinerea* F. Muell. ex Benth. (Myrtaceae) as a Source of 1,8-Cineole and Their Bioactivities. *Pharmaceuticals* **2011**, *4*, 1535–1550.
37. Franco, J.; Nakashima, T.; Franco, L.; Boller, C. Composição química e atividade antimicrobiana *in vitro* do óleo essencial de *Eucalyptus cinerea* F. Mull. Ex Benth., Myrtaceae, extraído em diferentes intervalos de tempo. *Rev. Bras. Farmacogn.* **2005**, *15*, 191–194.
38. Lee, Y.-S.; Kim, J.; Shin, S.-C.; Lee, S.-G.; Park, I.-K. Antifungal activity of Myrtaceae EOs and their components against three phytopathogenic fungi. *Flavour Fragr. J.* **2008**, *23*, 23–28.

39. Bossou, A.D.; Mangelinckx, S.; Yedomonhan, H.; Boko, P.M.; Akogbeto, M.C.; Kimpe, N.; Avlessi, F.; Sohounhloue, D.C.K. Chemical composition and insecticidal activity of plant EOs from Benin against *Anopheles gambiae* (Giles). *Parasit. Vectors* **2013**, *6*, 337.
40. Bossou, A.D.; Ahoussi, E.; Ruysbergh, E.; Adams, A.; Smagghe, G.; De Kimpe, N.; Avlessi, F.; Sohounhloue, D.C.K.; Mangelinckx, S. Characterization of volatile compounds from three Cymbopogon species and *Eucalyptus citriodora* from Benin and their insecticidal activities against Tribolium castaneum. *Ind. Crops Prod.* **2015**, *76*, 306–317.
41. Chagas, A.C.S.; Passos, W.M.; Prates, H.T.; Leitem, R.C.; Furlong, J.; Fortes, I.C.P. Acaricide effect of *Eucalyptus* spp. EOs and concentrated emulsion on *Boophilus microplus*. *Braz. J. Vet. Res. Ann. Sci.* **2002**, *39*, 247–253.
42. Gusmao, N.M.S.; Oliveira, J.V.; Navarro, D.M.A.F.; Dutra, K.A.; Silva, W.A.; Wanderley, M.J.A. Contact and fumigant toxicity and repellency of *Eucalyptus citriodora* Hook., *Eucalyptus staigeriana* F., *Cymbopogon winterianus* Jowitt and *Foeniculum vulgare* Mill. EOs in the management of *Callosobruchus maculatus* (FABR.) (Coleoptera: Chrysomelidae, Bruchinae). *J. Stored Prod. Res.* **2013**, *54*, 41–47.
43. Estanislau, A.A.; Barros, F.A.S.; Peña, A.P.; Santos, S.C.; Ferri, P.H.; Paula, J.R. Composição química e atividade antibacteriana dos óleos essenciais de cinco espécies de *Eucalyptus* cultivadas em Goiás. *Rev. Bras. Farmacogn.* **2001**, *11*, 95–100.
44. Macedo, I.T.F.; Bevilaqua, C.M.L.; Oliveira, L.M.B.; Camurça-Vasconcelos, A.L.F.; Vieira, L.S.; Amóra, S.S.A. Evaluation of *Eucalyptus citriodora* essential oil on goat gastrointestinal nematodes. *Rev. Bras. Parasitol. Vet.* **2011**, *20*. 223–227.
45. Maciel, M.V.; Morais, S.M.; Bevilaqua, C.M.L.; Silva, R.A.; Barros, R.S.; Sousa, R.N.; Sousa, L.C.; Brito, E.S.; Souza-Neto, M.A. Chemical composition of *Eucalyptus* spp. EOs and their insecticidal effects on *Lutzomyria longipalpis*. *Vet. Parasitol.* **2010**, *167*, 1–7.
46. Lima, J.K.A.; Albuquerque, E.L.D.; Santos, A.C.C.; Oliveira, A.P.; Araujo, A.P.A.; Blank, A.F.; Arrigoni-Blank, M.F.; Alves, P.B.; Santos, D.A.; Bacci, L. Biotoxicity of some plant EOs against the termite *Nasutitermes corniger* (Isoptera: Termitidae). *Ind. Crops Prod.* **2013**, *47*, 246–251.
47. Ribeiro, J.C.; Ribeiro, W.L.C.; Camurça-Vasconcelos, A.L.F.; Macedo, I.T.F.; Santos, J.M.L.; Paula, H.C.B.; Araujo Filho, J.V.; Magalhães, R.D.; Bevilaqua, C.M.L. Efficacy of free and nanoencapsulated *Eucalyptus citriodora* EOs on sheep gastrointestinal nematodes andtoxicity for mice. *Vet. Parasitol.* **2014**, *204*, 243–248.
48. Tomaz, M.A.; Costa, A.V.; Rodrigues, W.N.; Pinheiro, P.F.; Parreira, L.A.; Rinaldo, D.; Queiroz, V.T. Chemical composition and allelopathic activity of the *Eucalyptus* essential oil. *Biosci. J.* **2014**, *30*, 475–483.
49. Aguiar, R.W.S.; Ootani, M.A.; Ascencio, S.D.; Ferreira, T.P.S.; Santos, M.M.; Santos, G.R. Fumigant antifungal activity of *Corymbia citriodora* and *Cymbopogon nardus* EOs and citronellal against three fungal species. *Sci. World J.* **2014**, *2014*, 149–168.
50. Han, J.; Choi, B.R.; Lee, S.G.; Kim, S.I.; Ahn, Y.J. Toxicity of plant EOs to acaricide-susceptible and -resistant *Tetranychus urticae* (Acari: Tetranychidae) and *Neoseiulus californicus* (Acari: Phytoseiidae). *J. Econ. Entomol.* **2010**, *103*, 1293–1298.
51. Han, J.; Kim, S.I.; Choi, B.R.; Lee, S.G.; Ahn, Y.J. Fumigant toxicity of lemon *Eucalyptus* oil constituents to acaricide-susceptible and acaricide-resistant *Tetranychus urticae*. *Pest Manag. Sci.* **2011**, *67*, 1583–1588.
52. George, D.R.; Masic, D.; Sparagano, O.A.E.; Guy, J.H. Variation in chemical composition and acaricidal activity against *Dermanyssus gallinae* of four *Eucalyptus* EOs. *Exp. Appl. Acarol.* **2009**, *48*, 43–50.
53. Vera, S.S.; Zambrano, D.F.; Méndez-Sanchez, S.C.; Rodríguez-Sanabria, F.; Stashenko, E.E.; Luna, J.E.D. EOs with insecticidal activity against larvae of *Aedes aegypti* (Diptera: Culicidae). *Parasitol. Res.* **2014**, *113*, 2647–2654.
54. Olivero-Verbel, J.; Nerio, L.S.; Stashenko, E.E. Bioactivity against *Tribolium castaneum* Herbst (Coleoptera: Tenebrionidae) of *Cymbopogon citratus* and *Eucalyptus citriodora* EOs grown in Colombia. *Pest Manag. Sci.* **2010**, *66*, 664–668.
55. Olivero-Verbel, J.; Tirado-Ballestas, I.; Caballero-Gallardo, K.; Stashenko, E.E. EOs applied to the food act as repellents toward *Tribolium castaneum*. *J. Stored Prod. Res.* **2013**, *55*, 145–147.
56. Batish, D.R.; Singh, H.P.; Setia, N.; Kaur, S.; Kohli, R.K. Chemical composition and inhibitory activity of essential oil from decaying leaves of *Eucalyptus citriodora*. *Z. Naturforsch.* **2006**, *61*, 52–56.
57. Batish, D.R.; Singh, H.P.; Setia, N.; Kaur, S.; Kohli, R.K. Chemical composition and phytotoxicity of volatile essential oil from intact and fallen leaves of *Eucalyptus citriodora*. *Z. Naturforsch.* **2006**, *61*, 465–471.

58. Mulyaningsih, S.; Sporer, F.; Reichling, J.; Wink, M. Antibacterial activity of EOs from *Eucalyptus* and of selected components against multidrug-resistant bacterial pathogens. *Pharm. Biol.* **2011**, *49*, 893–899.
59. Lee, S.O.; Choi, G.J.; Jang, K.S.; Lim, H.K.; Cho, K.Y.; Kim, J. Antifungal activity of five plant EOs as fumigant against postharvest and soilborne plant pathogenic fungi. *Plant Pathol. J.* **2007**, *23*, 97–102.
60. Elaissi, A.; Rouis, Z.; Mabrouk, S.; Salah, K.B.H.; Aouni, M.; Khouja, M.L.; Farhat, F.; Chemli, R.; Harzallah-Skhiri, F. Correlation between chemical composition and antibacterial activity of EOs from fifteen *Eucalyptus* species. Growing in the Korbous and Jbel Abderrahman Arboreta (North East Tunisia). *Molecules* **2012**, *17*, 3044–3057.
61. Gilles, M.; Zhao, J.; An, M.; Agboola, S. Chemical composition and antimicrobial properties of EOs of three Australian *Eucalyptus* species. *Food Chem.* **2010**, *119*, 731–737.
62. Delaquis, P.J.; Stanich, K.; Girard, B.; Mazza, G. Antimicrobial activity of individual and mixed fractions of dill, cilantro, coriander and *Eucalyptus* EOs. *Int. J. Food Microbiol.* **2002**, *74*, 101–109.
63. Aref, S.P.; Valizadegan, O.; Farashiani, M.E. *Eucalyptus dundasii* Maiden essential oil, chemical composition and insecticidal values against *Rhyzopertha dominica* (F.) and *Oryzaephilus surinamensis* (L.). *J. Plant Prot. Res.* **2015**, *55*, 35–41.
64. Alzogaray, R.A.; Lucia, A.; Zerba, E.N.; Masuh, H.M. Insecticidal activity of EOs from eleven *Eucalyptus* spp. and two hybrids: lethal and sublethal effects of their major components on *Blattella germânica*. *J. Econ. Entomol.* **2011**, *104*, 595–600.
65. Toloza, A.C.; Lucia, A.; Zerba, E.; Masuh, H.; Picollo, M.I. *Eucalyptus* essential oil toxicity against permethrin-resistant *Pediculus humanus capitis* (Phthiraptera: Pediculidae). *Parasitol. Res.* **2010**, *106*, 409–414.
66. Ghnaya, A.B.; Hanana, M.; Amri, I.; Balti, H.; Gargouri, S.; Jamoussi, B.; Hamrouni, L. Chemical composition of *Eucalyptus erythrocorys* EOs and evaluation of their herbicidal and antifungal activities. *J. Pest Sci.* **2013**, *86*, 571–577.
67. Aref, S.P.; Valizadegan, O.; Farashiani, M.E. The Insecticidal Effect of Essential Oil of *Eucalyptus floribundi* Against Two Major Stored Product Insect Pests; *Rhyzoperth dominica* (F.) and *Oryzaephilus surinamensis* (L.). *J. Essent. Oil Bear. Plants* **2016**, *19*, 820–831.
68. Harkat-Madouri, L.; Asma, B.; Madani, K.; Said, Z.B.S.; Rigou, P.; Grenier, D.; Allalou, H.; Remini, H.; Adjaoud, A.; Boulekbache-Makhlouf, L. Chemical composition, antibacterial and antioxidant activities ofessential oil of *Eucalyptus globulus* from Algeria. *Ind. Crops Prod.* **2015**, *78*, 148–153.
69. Russo, S.; Cabrera, N.; Chludil, H.; Yaber-Grass, M.; Leicach, S. Insecticidal activity of young and mature leaves essential oil from *Eucalyptus globulus* Labill. against *Tribolium confusum* Jacquelin du Val (Coleoptera: Tenebrionidae). *Chi. J. Agric. Res.* **2015**, *75*, 375–379.
70. Yang, Y.; Choi, H.; Choi, W.; Clark, J.M.; Ahn, Y. Ovicidal and adulticidal activity of *Eucalyptus globulus* leaf oil terpenoids against *Pediculus humanus capitis* (Anoplura: Pediculidae). *J. Agric. Food Chem.* **2004**, *52*, 2507–2511.
71. Lee, B.; Choi, W.; Lee, S.; Park, B. Fumigant toxicity of EOs and their constituent compounds towards the rice weevil, *Sitophilus oryzae* (L.). *Crop Prot.* **2001**, *20*, 317–320.
72. Vilela, G.R.; Almeida, G.S.; D'Arce, M.A.B.R.; Moraes, M.H.D.; Brito, J.O.; Silva, M.F.G.F.; Silva, S.C.; Piedade, S.M.S.; Calori-Domingues, M.A.; Gloria, E.M. Activity of essential oil and its major compound, 1,8-cineole, from *Eucalyptus globulus* Labill., against the storage fungi *Aspergillus flavus* Link and *Aspergillus parasiticus* Speare. *J. Stored Prod. Res.* **2009**, *45*, 108–111.
73. Macedo, I.T.F.; Bevilaqua, C.M.L.; Oliveira, L.M.B.; Camurça-Vasconcelos, A.L.F.; Vieira, L.S.; Oliveira, F.R.; Queiroz-Junior, E.M.; Portela, B.G.; Barros, R.S.; Chagas, A.C.S. Ovicidal and larvicidal activity *in vitro* of *Eucalyptus globulus* EOs on *Haemonchus contortus*. *Rev. Bras. Parasitol. Vet.* **2009**, *18*, 62–66.
74. Yones, D.A.; Bakir, H.Y.; Bayoumi, S.A.L. Chemical composition and efficacy of some selected plant oils against *Pediculus humanus capitis* in vitro. *Parasitol Res.* **2016**, *115*, 3209–3218.
75. Mekonnen, A.; Yitayew, B.; Tesema, A.; Taddese, S. In Vitro Antimicrobial activity of essential oil of *Thymus schimperi*, *Matricaria chamomilla*, *Eucalyptus globulus*, and *Rosmarinus officinalis*. *Int. J. Microbiol.* **2016**, *2016*, 1–8.
76. Gupta, A.; Sharma, S.; Naik, S.N. Biopesticidal value of selected EOs against pathogenic fungus, termites, and nematodes. *Int. Biodeterior. Biodegrad.* **2011**, *65*, 703–707.

77. Pandey, A.; Chattopadhyay, P.; Banerjee, S.; Pakshirajan, K.; Singh, L. Antitermitic activity of plant EOs and their major constituents against termite *Odontotermes assamensis* Holmgren (Isoptera: Termitidae) of North East India. *Int. Biodeterior. Biodegrad.* **2012**, *75*, 63–67.
78. Pant, M.; Dubey, S.; Patanjali, P.K.; Naik, S.N.; Sharma, S. Insecticidal activity of *Eucalyptus* oil nanoemulsion with karanja and jatropha aqueous filtrates. *Int. Biodeterior. Biodegrad.* **2014**, *91*, 119–127.
79. Tyagi, A.K.; Malik, A. Antimicrobial potential and chemical composition of *Eucalyptus globulus* oil in liquid and vapour phase against food spoilage microorganisms. *Food Chem.* **2011**, *126*, 228–235.
80. Kumar, P.; Mishra, S.; Malik, A.; Satya, S. Compositional analysis and insecticidal activity of *Eucalyptus globulus* (family: Myrtaceae) essential oil against housefly (*Musca domestica*). *Acta Trop.* **2012**, *122*, 212–218.
81. Golestani, M.R.; Rad, M.; Bassami, M.; Afkhami-Goli, A. Analysis and evaluation of antibacterial effects of new herbal formulas, AP-001 and AP-002, against *Escherichia coli* O157:H7. *Life Sci.* **2015**, *135*, 22–26.
82. Tohidpour, A.; Sattari, M.; Omidbaigi, R.; Yadegar, A.; Nazemi, J. Antibacterial effect of EOs from two medicinal plants against Methicillin-resistant *Staphylococcus aureus* (MRSA). *Phytomedicine* **2010**, *17*, 142–145.
83. Fratini, F.; Casella, S.; Leonardi, M.; Pisseri, F.; Ebani, V.V.; Pistelli, L.; Pistelli, L. Antibacterial activity of EOs, their blends and mixtures of their main constituents against some strains supporting livestock mastitis. *Fitoterapia* **2014**, *96*, 1–7.
84. Vratnica, B.D.; Đakov, T.; Šuković, D.; Damjanović, J. Antimicrobial Effect of Essential Oil Isolated from *Eucalyptus globulus* Labill. from Montenegro. *Czech J. Food Sci.* **2011**, *29*, 277–284.
85. Derwich, E.; Benziane, Z.; Boukir, A. GC/MS analysis of volatile constituents and antibacterial activity of the essential oil of the leaves of *Eucalyptus globulus* in Atlas Median from Morocco. *Adv. Nat. Appl. Sci.* **2009**, *3*, 305–313.
86. Luis, A.; Duarte, A.; Gominho, J.; Domingues, F.; Duarte, A.P. Chemical composition, antioxidant, antibacterial and anti-quorumsensing activities of *Eucalyptus globulus* and *Eucalyptus radiata* essential oils. *Ind. Crops Prod.* **2016**, *79*, 274–282.
87. Marzoug, H.N.B.; Bouajila, J.; Ennajar, M.; Lebrihi, A.; Mathieu, F.; Couderc, F.; Abderraba, M.; Romdhane, M. *Eucalyptus* (*gracilis*, *oleosa*, *salubris*, and *salmonophloia*) EOs: Their chemical composition and antioxidant and antimicrobial activities. *J. Med. Food* **2010**, *13*, 1005–1012.
88. Lucia, A.; Gonzalez, A.P.; Seccacini, E.; Licastro, S.; Zerba, E.; Masuh, H.M. Larvicidal effect of *Eucalyptus grandis* essential oil and turpentine and their major components on *Aedes aegypti* larvae. *J. Am. Mosq. Control Assoc.* **2007**, *23*, 299–303.
89. Cerceau, C.I.; Barbosa, L.C.A.; Filomeno, C.A.; Alvarenga, E.S.; Demuner. A.J.; Fidencio, P.H. An optimized and validated ^{1}H NMR method for the quantification of α–pinene in essentials oils. *Talanta* **2016**, *150*, 97-103.
90. Proenza, Y.G.; Álvarez, R.Q.; Tamayo, Y.V.; Saavedra, M.A.; García, Y.S.; Espinosa, R.H. Chemical composition and antibacterial activity of the essential oil from *Eucalyptus pellita* F. Muell. *J. Med. Plants Res.* **2013**, *7*, 1979–1983.
91. Camara, B.; Dick, E.; Sako, A.; Kone, D.; Kanko, C.; Boye, M.A.D.; Ake, S.; Anno, A. Lutte biologique contre *Deightoniella torulosa* (Syd.) Ellis, par l'application des huiles essentielles d'*Eucalyptus platyphylla* F. Muell. et de *Melaleuca quinquenervia* L. *Phytothérapie* **2010**, *8*, 240–244.
92. Sartorelli, P.; Marquioreto, A.D.; Amaral-Baroli, A.; Lima, M.E.L.; Moreno, P.R.H. Chemical composition and antimicrobial activity of the EOs from two species of *Eucalyptus*. *Phytotherapy* **2007**, *21*, 231–233.
93. Gillij, Y.G.; Gleiser, R.M.; Zygadlo, J.A. Mosquito repellent activity of EOs of aromatic plants growing in Argentina. *Bioresour. Technol.* **2008**, *99*, 2507–2515.
94. Bett, P.K.; Deng, A.L.; Ogendob, J.O.; Kariuki, S.T.; Kamatenesi-Mugisha, M.; Mihale, J.M.; Torto, B. Chemical composition of *Cupressus lusitanica* and *Eucalyptus saligna* leaf essential oils and bioactivity against major insect pests of stored food grains. *Ind. Crops Prod.* **2016**, *82*, 51–62.
95. Safaei-Ghomi, J.; Batooli, H. Chemical composition and antimicrobial activity of the volatile oil of *Eucalyptus sargentii* Maiden cultivated in central Iran. *Int. J. Green Pharm.* **2010**, *4*, 174–177.
96. Baptista. E.B.; Zimmermann-Franco, D.C.; Lataliza, A.A.B.; Raposo, N.R.B. Chemical composition and antifungal activity of essential oil from *Eucalyptus smithii* against dermatophytes. *Rev. Soc. Bras. Med. Trop.* **2015**, *48*, 746–752.

97. Ribeiro, W.L.C.; Camurça-Vasconcelos, A.L.F.; Macedo, I.T.F.; Santos, J.M.L.; Araujo-Filho, J.V.; Ribeiro, J.C.; Pereira, V.A.; Viana, D.A.; Paula, H.C.B.; Bevilaqua, C.M.L. In vitro effects of *Eucalyptus staigeriana* nanoemulsion on *Haemonchus contortus* and toxicity in rodents. *Vet. Parasitol.* **2015**, *212*, 444–447.
98. Ribeiro, W.L.C.; Macedo, I.T.F.; Santos, J.M.L.; Oliveira, E.F.; Camurça-Vasconcelos, A.L.F.; Paula, H.C.B.; Bevilaqua, C.M.L. Activity of chitosan-encapsulated *Eucalyptus staigeriana* essential oil on *Haemonchus contortus*. *Exp. Parasitol.* **2013**, *135*, 24–29.
99. Macedo, I.T.F.; Bevilaqua, C.M.L.; Oliveira, L.M.B.; Camurça-Vasconcelos, A.L.F.; Vieira, L.S.; Oliveira, F.R.; Queiroz-Junior, E.M.; Tomé, A.R.; Nascimento, N.R.F. Anthelmintic effect of *Eucalyptus staigeriana* essential oil against goat gastrointestinal nematodes. *Vet. Parasitol.* **2010**, *173*, 93–98.
100. Alitonou, G.; Avlessi, F.; Wotto, V.D.; Ahoussi, E.; Dangou, J.; Sohounhloué, D.C.K. Composition chimique, propriétés antimicrobiennes et activités sur les tiques de l'huile essentielle *d'Eucalyptus tereticornis* Sm. *C. R. Chim.* **2004**, *7*, 1051–1055.

© 2016 by the authors. Submitted for possible open access publication under the terms and conditions of the Creative Commons Attribution (CC-BY) license (http://creativecommons.org/licenses/by/4.0/).
